# Partial Hydrogenation of Waste Cooking Oil Biodiesel Catalyzed by Iron Oxide/Nickel Nanoparticles Supported on Steel Slags

**DOI:** 10.1021/acsomega.5c05619

**Published:** 2025-11-05

**Authors:** Maria Stella Leone, Piero Mastrorilli, Ernesto Mesto, Emanuela Schingaro, Roberto Comparelli, Carlo Porfido, Maria Michela Dell’Anna

**Affiliations:** † Department of Civil, Environmental, Land, Building Engineering and Chemistry, 18951 Politecnico di Bari, 4 via Orabona, Bari 70125, Italy; ‡ Department of Geomineralogy, 9295 University of Bari, 4 via Orabona, Bari I-70125, Italy; § Institute for Physical and Chemical Processes—IPCF, National Research Council, 4 via Orabona, Bari I-70125, Italy; ∥ Department of Soil, Plant and Food Sciences, University of Bari, 165/A Via Amendola, Bari 70126, Italy

## Abstract

Biodiesel, produced mainly through transesterification of vegetable oils or animal fats, is a promising alternative to fossil fuels due to its biodegradability and reduced environmental impact. Biodiesel synthesized from waste cooking oil (WCO) is even more appealing from an environmental sustainability point of view. However, the crude WCO biodiesel is not immediately suitable as a fuel due to inherent limitations such as poor oxidative stability and suboptimal cold flow properties. To overcome these issues, an upgrading reaction must be carried out, which consists of a partial hydrogenation of the polyunsaturated chains of FAMEs aiming at converting polyunsaturated compounds to monounsaturated ones while avoiding fully saturated products. This study introduces a strategic modification of waste steel slag (SS) to obtain a new catalyst effective in the WCO biodiesel upgrading step. A bimetallic Fe–Ni system onto SS was synthesized aiming at combining the stability and basic properties of the support with the high catalytic activity of the metals. Two methods for partial hydrogenation were employed: the conventional partial hydrogenation method with molecular hydrogen and the catalytic transfer hydrogenation using NaBH_4_. Both methods utilized heterogeneous nickel–iron oxide-based catalysts supported onto SS leading to a WCO biodiesel mixture rich in C18:1 chains.

## Graphical abstract

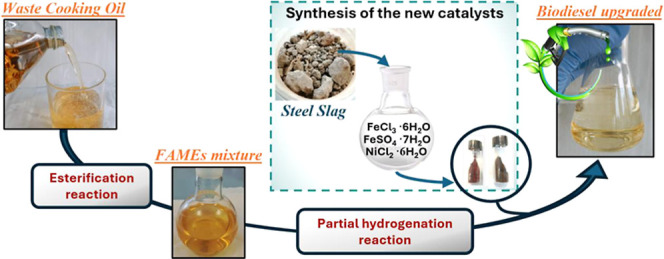

## 1 Introduction

Greenhouse gas emissions leading to global warming and consequently climate change are the most pressing and alarming environmental problems to be addressed in the current century.^1^ The combustion of fossil fuels is a major cause of climate change. Therefore, new sources of energy must be exploited to break the dependence on fossil and nonrenewable sources. Biomass, or any organic material that comes from trees, plants, and agricultural and organic waste, is a very promising renewable source of energy and can result in a variety of valuable products, such as chemicals, biopolymers, biomaterials, and biofuels.^2^ Among the various fuels, biodiesel is a very promising fuel, derived from renewable sources such as vegetable oils and animal fats, that can replace conventional fossil fuels. Technically, biodiesel is a mixture of alkyl esters of fatty acids, produced through the transesterification of oils or fats in the presence of short-chain alcohols, such as methanol or ethanol, and a catalyst, giving glycerin as a byproduct.^3^ Biodiesel is biodegradable, nontoxic, and able to reduce engine emissions^4^ such as unburned hydrocarbons, particulate matter, carbon monoxide, carbon dioxide, sulfur oxide, and polycyclic aromatic hydrocarbons. Meanwhile, it is safer than fossil fuels to be stored and handled and can be easily produced in large quantities. Biodiesel is classified into four groups, called generations, depending on the nature of the feedstock used for its synthesis.^5^ *First-generation biodiesel* is prepared from edible oils deriving from agricultural materials such as cereals and sugar canes, *second-generation biodiesel* derives from nonedible oils of dedicated cultivars, *third-generation biodiesel* derives from waste vegetable oils, and *fourth-generation biodiesels* deriving from algae. In this work, the studied biodiesel belongs to the third generation being obtained starting from waste cooking oil (WCO).

It is estimated that over 15 million tons of used vegetable oil are produced worldwide each year, with nearly 1 million tons coming from the European Union (EU) alone.^6^ The increasing production of WCO creates serious disposal issues. In most cases, WCO is discarded as waste and improperly landfilled in garbage, soil, or sewerage systems, leading to water treatment problems such as reduced sewer diameters and blockages in wastewater treatment plant infrastructure.^7^ Therefore, appropriate management of WCO leads not only to environmental benefits but also to economic advantages. The use of WCO as a raw material for biodiesel production makes the process very appealing from an economic point of view due to its low cost. The WCO price is two to three times cheaper than vegetable oils; indeed, the usage of waste edible oils can reduce biodiesel production costs by 60–90%.^8^ The properties of WCO are different from fresh oil due to physical and chemical changes that occur during frying, such as oxidation and hydrolysis reactions. WCO has a higher content of free fatty acids and water than fresh oil.^9^ Although several techniques have been used to obtain biofuel from WCO, such as gasification or pyrolysis, the most widely used process is the catalytic transesterification of WCO in methanol to synthesize biodiesel due to its economical and environmentally friendly procedure.^10^ The crude biodiesel is not immediately usable as a fuel due to inherent limitations, such as poor oxidative stability and suboptimal cold flow properties.^11^ To overcome these issues, an upgrading reaction must be carried out, which consists of a partial hydrogenation reaction of the polyunsaturated chains of FAMEs with the objective of converting polyunsaturated compounds to the monounsaturated ones while avoiding obtaining a fully saturated product. Partial hydrogenation of FAMEs can be conducted by employing different hydrogen sources, such as molecular hydrogen (H_2_, conventional partial hydrogenation), or hydrogen in situ generated by catalytic systems employing hydrides, formic acid, branched alcohols, polyols, and others (catalytic transfer hydrogenation, CTH).^12^ The use of sodium borohydride as a hydrogen donor would provide a new way for hydrogenation of oils and fats due to its environmentally friendly properties and its safe handling.^13^ Most catalysts used for biodiesel upgrading are constituted of metal particles dispersed on porous supports made of carbon, silicon, or aluminum oxides.^12^ The metals typically encountered in catalytic systems are Pt,^14^ Pd,^15^ and Ni.^16^ Nickel-based supported catalysts are commonly used due to their lower cost compared to noble metal-based catalysts.^17^ In the partial hydrogenation reaction of FAMEs, nickel catalysts play a particularly important role. However, monometallic Ni-based catalysts have demonstrated lower catalytic activity than bimetallic systems,^18^ failing to achieve conversion levels that would significantly improve biodiesel properties. Recent studies have shown that the addition of other metals in combination with Ni, such as noble metals,^19^ other transition metals,^20^ alkali metals,^21^ and alkaline earth metals,^22^ used principally in steam reforming reactions, enhances the catalytical activity of the whole system. Among them, different catalytic systems based on Ni nanoparticles supported onto iron oxides^23^ have been reported for various reactions, such as hydrocarbon reforming,^24^ hydrodeoxygenation,^25^ and hydrogenation.^26,27^ In this work, bimetallic catalysts based on nickel and iron oxides nanoparticles supported onto steel slags were used. Steel slag (SS) is a solid byproduct generated during steel production and represents the primary solid waste of this process, accounting for 15–20% of the total output.^28^ The global annual output of WCO surpasses 190 million metric tons, with the EU accounting for approximately 7.7 million tons per year.^29^ However, a significant portion of it is often buried or stored outdoors, occupying large areas of land and contributing to soil and groundwater pollution. Moreover, the presence of heavy metals in SS can lead to the release of pollutants into the environment, posing risks to ecosystems and human health.

Currently, SS is mainly used in cement production, construction materials, and road paving.^30^ An innovative application is the use of steel slag as a catalytic support,^31^ promoting waste recycling while reducing both catalytic conversion costs and environmental impact. This strategy aligns with a circular development model, representing a beneficial approach to preventing resource depletion and waste accumulation, ultimately transforming a problematic byproduct into a valuable resource for energy and economic sustainability.

## 2 Experimental Section

### 2.1 Materials

All chemicals and solvents were purchased from commercial sources and used as received. Tap water was deionized before use with ionic exchange resins (Millipore). Waste steel slags (SS) employed in the present study were derived from the secondary slag by the steel factory “ILVA” (Taranto, Italy), and they remained in an open-air disposal site for more than 40 years. WCO was obtained from domestic waste. FAMEs were prepared from WCO through a transesterification reaction according to the procedure reported by Fiore et al.^16^

### 2.2 General Consideration

Fourier transform infrared (FT-IR) spectra were recorded on a Jasco FT-IR 4200 spectrometer. Surface morphology was investigated on a selected piece of sample considered to be representative of the material. A FE-SEM Zeiss Sigma 300 VP (Zeiss Oberkochen, Germany) equipped with an energy-dispersive spectrometer (EDS) C-MaxN SDD (Oxford Instruments, Oxford, U.K.) with an active area of 20 mm^2^ (Oxford Instruments, Oxford, U.K.) was used to perform analysis on the selected samples. The EDS spectrometer was calibrated using MAC standards (Micro-Analysis Consultants Ltd., United Kingdom) for elemental analysis.

X-ray powder diffraction data were collected in air using a PANalytical Empyrean X-ray diffractometer with Bragg–Brentano geometry, large beta filter-nickel, detector (PIXcel3D), and Cu Kα radiation, operating at 40 kV/40 mA. The X-ray data were collected in the 2θ range 10–90° (step size 0.026, scan step time 996.54 s). The diffraction patterns were processed using PANalytical B.V. software HIGHScore Plus version 3.0e. To determine the crystallite size of the identified phases, LeBail analysis was performed using Profex version 3.5.0.

Rietveld refinement^32,33^ is a computational technique widely used in crystallography to extract detailed structural information from powder diffraction data. By iteratively optimizing a calculated diffraction pattern to align with experimental data, this method refines instrumental and crystallographic parameters, yielding a precise structural model.^33^

In this study, Rietveld analysis was conducted using Profex software (v. 5.3.0), which provides a graphical interface for the BGMN refinement program.^32^ The refinement process involved the optimization of parameters such as scale factors, cell parameters, crystallite size distribution, microstrain, preferential orientation, and thermal parameters. Initial parameter values were sourced from crystallographic databases. Peak profile modeling utilized a Lorentzian function to represent the diffraction peaks, incorporating three primary contributions:

1 Instrumental function (G): describes the contribution of instrumental factors to peak broadening.
2 Sample function (P): accounts for broadening caused by crystallite size and microstrain.
3 Wavelength distribution function (D): reflects the spread of the X-ray wavelength used during the experiment.

The iterative refinement adjusted these parameters through numerical optimization, specifically employing least-squares minimization to minimize discrepancies between the experimental and calculated diffraction patterns. This approach allowed for the accurate separation of peak broadening effects to derive an accurate quantification of crystallite size of the occurred crystal phases.

The quality of the fit was evaluated using statistical indicators such as the weighted Rietveld factor (*R*_wp_) and reduced chi-squared (χ^2^). The final output included refined structural parameters and precise quantitative estimates of crystallite sizes. Transmission electron microscopy (TEM) analysis was performed by means of a JEOL JEM-1011 (JEOL, Akishima, Tokyo, Japan) microscope operating at 100 kV. The TEM samples were prepared by casting a drop of catalyst methanol dispersion onto a carbon-hollowed TEM grid. Gas chromatography analyses were carried out using an HP 6890 instrument equipped with a flame ionization detector and a capillary column coated with a cyanopropyl stationary phase (DB-FastFAME, 90 m × 0.25 mm × 0.25 μm). Elemental analyses on the catalysts were carried out with a portable energy-dispersive X-ray fluorescence spectrometer (P-ED-XRF, NITON XL3 t, Thermo Scientific, Waltham, USA) equipped with an Ag collimator source (50 KeV and 40 μA) and a large SSD detector (energy resolution <160 eV @Mn- Kα).

### 2.3 Catalyst Preparation

#### 2.3.1 Synthesis of **Fe-SS**

FeCl_3_·6H_2_O (6.0 mmol, 1.6220 g) and FeSO_4_·7H_2_O (3.0 mmol, 0.8341 g) were dissolved in deionized water (130 mL), obtaining an orange-brown solution, to which was added SS (2.08 g) as a dark brown solid. The resulting mixture was left under stirring at 35 °C for 6 h to give a colorless water phase and a reddish-brown precipitate referred to as **Fe-SS**. After centrifugation (4000 rpm × 10 min), the solid was removed from the supernatant solution, washed with deionized water (3 × 3 mL), and dried overnight at 85 °C.

$$Yield=2.3344\;g.{Fe\;\%}_{w}=38.12\pm 0.38$$

#### 2.3.2 Synthesis of **Ni/Fe-SS**

**Fe-SS** (0.82 g) was added to the green solution obtained by dissolving NiCl_2_·6H_2_O (3.45 mmol, 0.732 g) in 20 mL of deionized water and stirring at 65 °C overnight. Afterward, the whole mixture made of a light-blue water phase and a dark brown solid was put in an oven at 85 °C overnight until total water evaporated. The resulting solid (1.2614 g) was calcined at 300 °C for 30 min under an initial dihydrogen pressure of 5 bar, yielding a slightly magnetic dark red solid material (1.1579 g), referred to as **Ni/Fe-SS**. Elemental analyses: Fe %_w_ = 26.42 ± 0.54. Ni %_w_ = 11.24 ± 0.78.

#### 2.3.3 Synthesis of **Ni/Fe-SSb**

**Fe-SS** (0.82 g) was added to the green solution obtained by dissolving NiCl_2_·6H_2_O (3.45 mmol, 0.732 g) in 20 mL of deionized water and left stirring at 65 °C for 3 h. Afterward, few drops of NaOH (0.50 M) solution were added to the whole mixture until the water phase became colorless. After 1 h of stirring, the system was centrifuged, the liquid phase was removed with a glass pipet, and the solid was dried at 85 °C overnight, yielding a dark gray material (1.2228 g). 0.6759 g of the dark gray material was calcined at 300 °C for 30 min under an initial dihydrogen pressure of 5 bar, yielding a strongly magnetic black solid (0.6307 g), referred to as **Ni/Fe-SSb**. Elemental analyses: Fe %_w_ = 22.68 ± 0.16. Ni %_w_ = 13.27 ± 0.16.

### 2.4 Catalytic Test: Partial Hydrogenation of FAMEs under Dihydrogen

**Ni/Fe-SS** or **Ni/Fe-SSb** (50 mg) was added to a solution of biodiesel (125 mg) in methanol (5.0 mL) in a 50 mL stainless-steel reactor. The whole mixture was put under dihydrogen (5 bar) at 70 °C and left stirring for an appropriate amount of time. The progress of the reaction was monitored by GLC. At the end of the reaction, the catalyst was removed from the liquid phase and washed with methanol (3 × 5 mL). Then, it was dried under vacuum, weighed, and employed for subsequent catalytic runs, by using a proper amount of fresh reagent, for keeping steady the metal/substrate molar ratio.

### 2.5 Catalytic Test: Partial Hydrogenation of FAMEs with NaBH_4_

**Ni/Fe-SS** or **Ni/Fe-SSb** (50 mg) and NaBH_4_ (20 mg, 0.53 mmol) were added to a solution of biodiesel (125 mg) in methanol (5.0 mL) in a 25 mL three-necked round flask, equipped with a magnetic stirrer and a gas bubbler. The whole mixture was left stirring at room temperature (RT) for the appropriate amount of time. The progress of the reaction was monitored by GLC. At the end of the reaction, the catalyst was removed of the liquid phase and washed with deionized water (3 × 5 mL) and methanol (3 × 5 mL). Then, it was dried under vacuum, weighed, and used for subsequent catalytic runs.

## 3 Results

### 3.1 Synthesis and Characterization of the Iron Modified Support **Fe-SS**

The structural and chemical properties of the steel slags used in the present work have been already discussed in detail.^34^ The SS alkaline feature was revealed by the high pH value (>12) of the supernatant water phase mixed for 15 min with SS in 1/2.5 (SS/H_2_O) mass ratio, being SS constituted mainly of metal oxides, carbonates, silicates, aluminosilicates, and hydroxides.

Prior to coat SS with Ni catalytic centers, steel slags were treated with a Fe^2+^/Fe^3+^ (1/2 molar ratio) solution aiming at obtaining Fe_3_O_4_ deposited onto the inorganic material, for rendering the latter a highly magnetic support.^35^ This treatment had also the scope to enhance the catalytic activity of the subsequently deposited Ni active species.^17^ In fact, many studies have reported a synergic effect between the Fe^2+^/Fe^3+^ system and another metal center, resulting in an improving of the activity of the bimetallic catalyst compared to the monometallic one.^27^ Following the precipitation method under alkaline conditions,^36^ SS were mixed with a solution of Fe^2+^/Fe^3+^, avoiding the addition of an external base, due to the strong basic features of SS. In fact, this direct mixing led to the precipitation of iron hydroxides onto the SS surface without the addition of sodium hydroxide or ammonia, which, on the contrary, was mandatory in other reported works.^37^

The iron-modified SS support, **Fe-SS**,^38^ was used as the starting matrix to be covered by the Ni catalyst. XRF analysis (Table 1) shows that the **Fe-SS** matrix contained high quantities of Fe (38.12 ± 0.38%_w_), while the amount of Ca (6.77 ± 0.05%_w_) decreased with respect to pure steel slags (31.22 ± 1.8%_w_), suggesting the replacement of calcium cations for iron ions during treatment of SS with iron salt solution to give **Fe-SS**. After deposition, **Fe-SS** solution showed a slightly acidic pH (pH 5 ÷ 6) because of calcium oxide/hydroxide dissolution. In any case, XPS analyses carried out onto SS prior to the addition of iron salts revealed that the whole quantity of iron present in pristine SS was not available for catalytic purposes, being in the *core* part of the material, as reported and extensively discussed by Derobertis et al.^34^

**Table 1:** Elemental Analysis of the Investigated Steel Slags and the **Fe-SS** Matrix Carried out by XRF Analysis

element	%_w_ in steel slag	%_w_ in Fe-SS
Si	3.8 ± 0.2	2.97 ± 0.3
Ca	31.2 ± 1.8	6.77 ± 0.05
Mn	4.7 ± 0.4	3.87 ± 0.08
Fe	18.8 ± 1.4	38.12 ± 0.38

The IR spectrum of **Fe-SS** (Figure S1) shows a broad band at 3430 cm^–1^ belonging to the stretching vibration of OH functional groups, a sharp signal at 1620 cm^–1^ attributable to −OH bending vibrations, and small intensity peaks at 1411 cm^–1^ and 700 cm^–1^ typical for the presence of carbonates.^39^ The peak at 1127 cm^–1^ is attributable to the siloxane group Si–O–Si stretching vibrations.^40,41^ The FT-IR band at 970 cm^–1^ can be assigned to Si–OH stretching vibrations. These peaks are consistent with the presence of quartz and zeolites revealed by the XRD analysis. The peaks at 600, 670, and 700 cm^–1^ correspond to Fe–O stretching in iron oxides.^42,43^

FE-SEM analysis showed that **Fe-SS** is composed of particles with a spherical morphology beside the presence of some needles (Figure S2A). EDX (energy-dispersive X-ray) mapping (Figure S2) revealed a related elemental homogeneous distribution of Fe and Si together with O. On the contrary, the distribution of Mn and Mg, which are associated with each other, was rather irregular, with their concentrations being higher in certain areas compared to others. Moreover, the distribution of sulfur was correlated to that of calcium, likely due to the presence of calcium sulfate of needle-like morphology, as confirmed by XRD analyses (vide infra). Traces of chloride and phosphorus were also detected by EDX analysis.

The XRD diffractogram of **Fe-SS** (Figure S3) showed the presence of wüstite (FeO), quartz (SiO_2_), zeolite (Na­(AlSi_2_O_6_)·H_2_O), and magnesite (MgCO_3_), along with a significant amount of amorphous compounds, as suggested by the low level of signal/noise ratio in the diffractogram. Deeper analysis carried out on a material like **Fe-SS** revealed the presence of a big amount of ferrihydrite covering the surface of SS.^38^ By comparing the XRD diffractograms of SS^34^ and **Fe-SS**, the latter highlighted the disappearance of the signals related to the calcium-containing minerals, confirming the results obtained by XRF elemental analyses, which proved the drastic reduction of the calcium amount after iron deposition onto SS.

### 3.2 Synthesis and Characterization of the Catalysts **Ni/Fe-SS** and **Ni/Fe-SSb**

The Ni covering procedure onto **Fe-SS** was carried out in two different ways: impregnation or precipitation, respectively (path a and path b, Figure 1):

**Figure 1:**
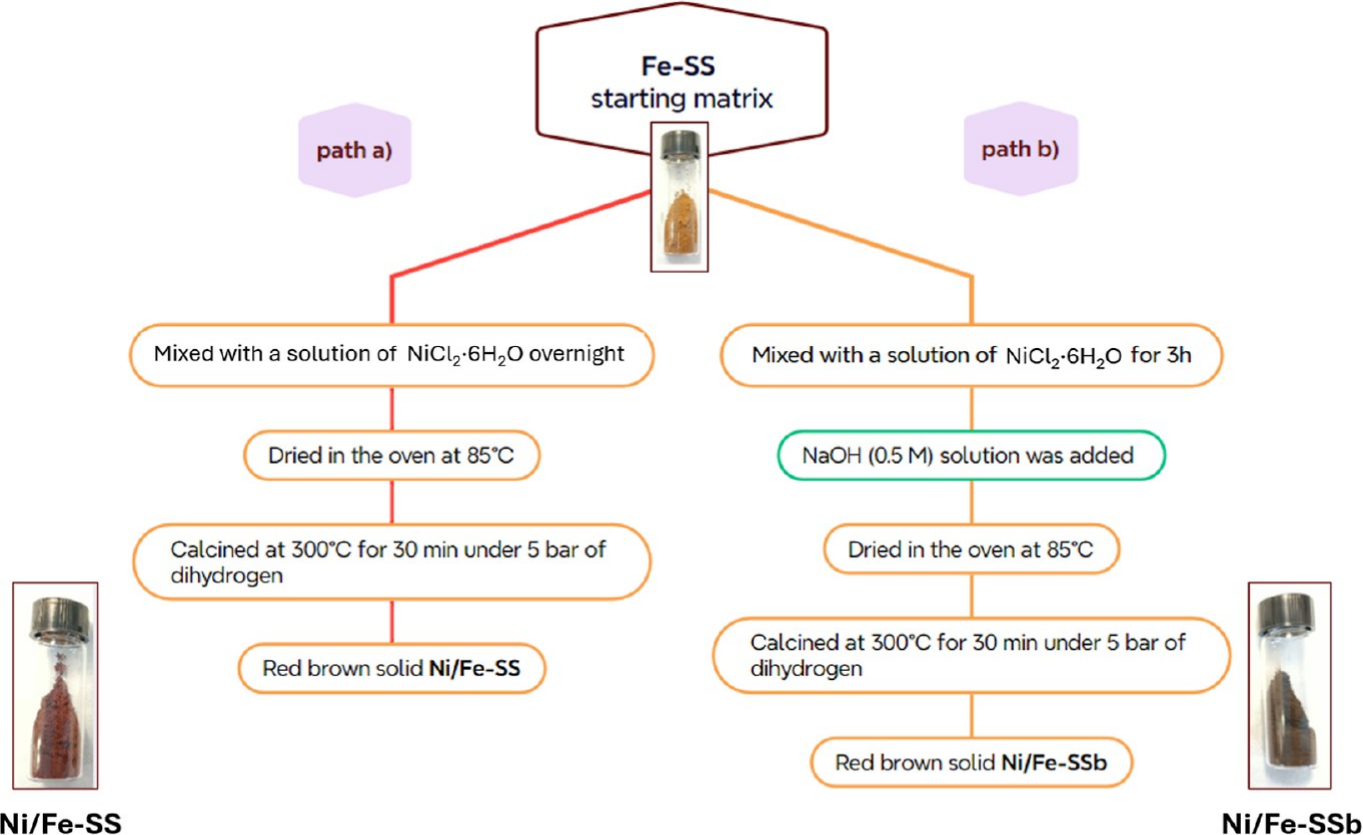
Synthesis of **Ni/Fe-SS** and **Ni/Fe-SSb**.

With the impregnation method, the pH value of the supernatant water solution (pH 5÷6) was not basic enough to cause the quantitative precipitation of Ni­(OH)_2_ onto **Fe-SS**, thus the solvent was evaporated under air at 85 °C, leaving Ni­(II) species onto the **Fe-SS** surface, before being calcined at 300 °C under hydrogen (5 bar) to give the **Ni/Fe-SS** catalyst (path a, Figure 1). With the precipitation method, a NaOH (0.50 M) solution was added to the mixture containing **Fe-SS** and dissolved nickel salt, causing the precipitation of nickel hydroxide onto **Fe-SS**. Afterward, the whole solid material was separated from the liquid phase and calcined at 300 °C under H_2_ (5 bar) to yield **Ni/Fe-SSb** (path b, Figure 1).

#### 3.2.1 Characterization of **Ni/Fe-SS**

XRF elemental analysis (Table 2) of **Ni/Fe-SS** confirmed the presence of Ni (11.24 ± 0.78%_w_) and highlighted the percent decreasing of the other elements with respect to **Fe-SS** (Table 1) due to Ni deposition.

**Table 2:** Elemental Composition of the **Ni/Fe-SS** Catalyst Assessed by XRF Analysis

element	%_w_ in Ni/Fe-SS
Si	2.06 ± 0.07
Ca	3.75 ± 0.05
Mn	2.18 ± 0.02
Fe	26.42 ± 0.54
Ni	11.24 ± 0.78

The FT-IR spectrum of **Ni/Fe-SS** (Figure 2) showed several distinct signals after thermal treatment, such as the broad band at 3430 cm^–1^, the peak at 1620 cm^–1^, and the signal at 810 cm^–1^ ^44^ belonging to adsorbed water and vibrations of O–H features,^45^ some of them already observed in the **Fe-SS** FT-IR spectrum (Figure S1);^46^ the absorption peaks observed between 1154 and 1000 cm^–1^, with distinct contributions at 1150, 1100, and 1020 cm^–1^, are characteristic of asymmetric Si–O–T stretching vibrations, where T represents either Si or Al,^47^ suggesting the presence of zeolite-like or silicate-based phases in the sample. These peaks overlap with the typical vibrations ν_sym_Al–O–H and ν_asym_Al–O–H modes (specifically 1150 and 1100 cm^–1^). The peaks at 670 and 720 cm^–1^ are associated with Fe–O stretching vibrations, consistent with the presence of hematite revealed by XRD analysis (vide infra^42^).

**Figure 2:**
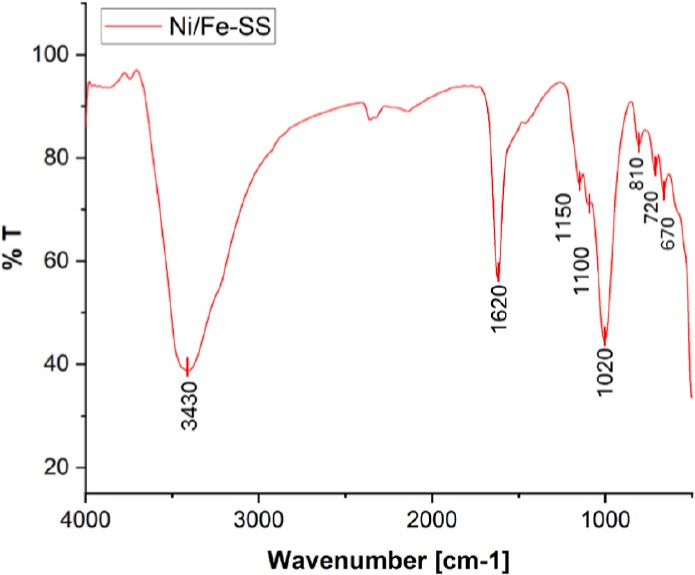
FT-IR spectrum of **Ni/Fe-SS**.

The FE-SEM image of **Ni/Fe-SS** (Figure 3A,B) reported a nanostructured material onto which all detected elements, including Ni and Fe, were homogeneously distributed (EDX maps, Figure 3). The presence of Cl could be justified on the basis of the nickel salt (NiCl_2_) employed for the preparation of the new catalyst by an impregnation method.

**Figure 3:**
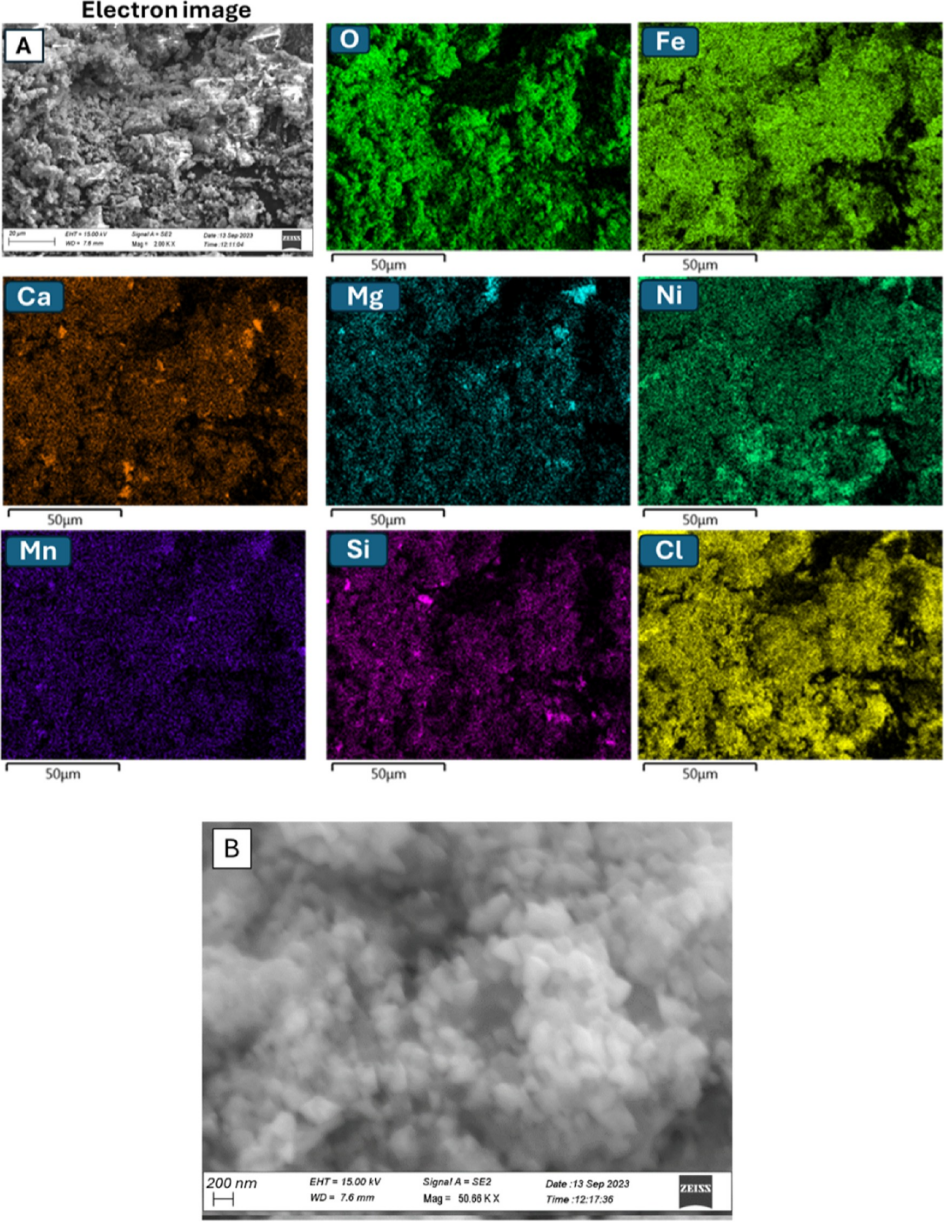
(A) FE-SEM image and corresponding EDX maps and (B) FE-SEM image at a different magnification of **Ni/Fe-SS**.

The X-ray diffraction pattern of the **Ni/Fe-SS** catalyst (Figure 4) revealed the presence of multiple crystalline phases. The most intense peak is attributed to the Dawsonite phase (NaAlCO_3_(OH)_2_), indicating the significant presence of this compound in the material. Several well-defined reflections associated with hematite (α-Fe_2_O_3_) confirmed the crystalline nature of the iron-based phase deposited on the steel support.

**Figure 4:**
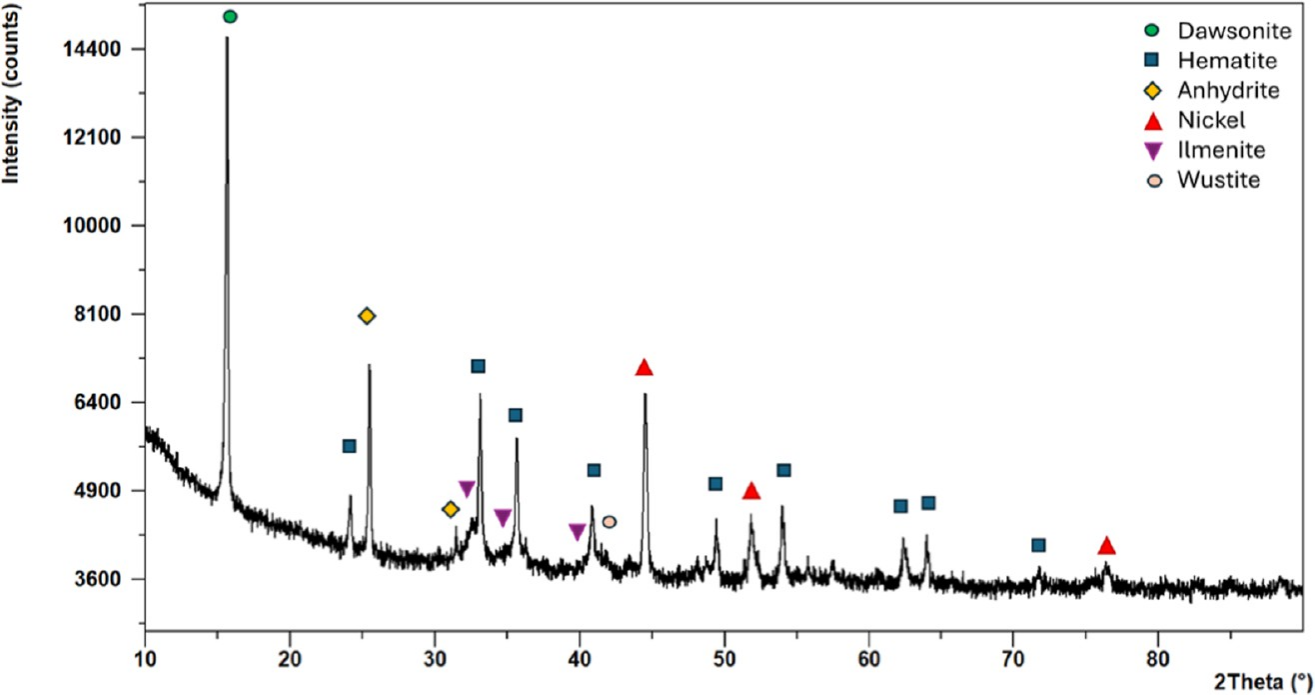
XRD diffractogram of **Ni/Fe-SS**.

Characteristic reflections of metallic nickel (Ni) are also clearly visible, indicating the successful deposition of nickel as the active metal phase. Additional signals corresponding to anhydrite (CaSO_4_), and ilmenite (FeTiO_3_), suggest the presence of secondary phases, which likely originated during the synthesis process or subsequent thermal treatment. On the contrary, wüstite (FeO) was already present in the pristine SS support. Thus, the diffraction pattern confirms the presence of both metallic Ni and iron oxide (hematite) and provides valuable insights into the structural interactions among the components of the catalytic system.

The diffractometric data were further analyzed to estimate the average crystallite size of the nickel and iron oxides nanoparticles by means of peak profile analysis through Rietveld refinement.^32^ The calculated average crystallite size of Ni was 54.2 ± 1.3 nm, indicating the formation of metallic nanocrystalline nickel in the new material, which could be advantageous from the catalytic activity point of view.^48^ Also, hematite was deposited in the form of nanoparticles with an average crystallite size of 73.2 ± 4.5 nm.

The presence of nanoparticles on **Ni/Fe-SS** was confirmed by TEM images (Figure 5), showing irregular aggregates featuring regions of very high electron density (dark areas) and less dense regions (lighter areas). The nanoparticles have variable sizes (ranging from 50 to 70 nm in diameter), and some of them are spherical or distinct cubic-shaped crystals, while others exhibit a more amorphous structure.

**Figure 5:**
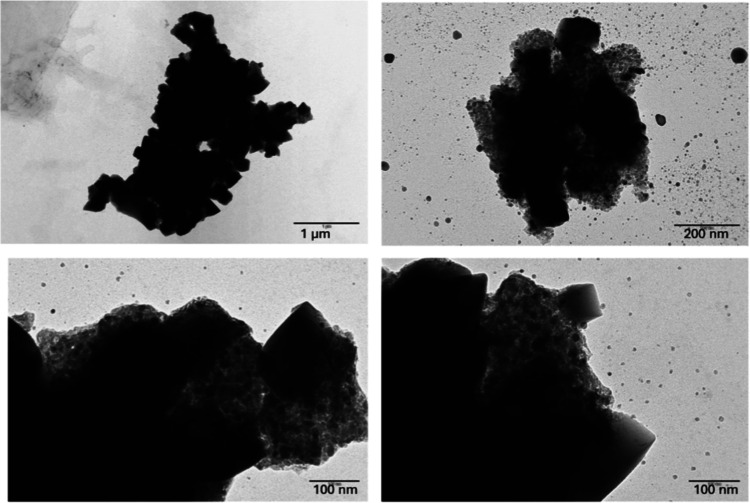
TEM images of **Ni/Fe-SS** at different magnifications.

#### 3.2.2 Characterization of **Ni/Fe-SSb**

The black highly magnetic solid **Ni/Fe-SSb** presented almost the same elemental composition of **Ni/Fe-SS**, as revealed by XRF analyses (Table 3).

**Table 3:** Elemental Composition of the **Ni/Fe-SSb** Catalyst

element	%_w_ in Ni/Fe-SSb
Si	1.92 ± 0.09
Ca	4.52 ± 0.07
Mn	2.38 ± 0.03
Fe	22.68 ± 0.16
Ni	13.27 ± 0.16

The FT-IR spectrum (Figure 6) showed a very intense band in the range of 3100–3500 cm^–1^ due to the O–H stretching of metal hydroxides, as expected due to the addition of sodium hydroxide during preparation. The absorption band at 1628 cm^–1^ could be attributable to the O–H bending vibration mode,^45^ the absorption band at 1550–1410 cm^–1^ corresponded to the asymmetric stretching vibration mode of the carbonate anion group (CO_3_^2–^),^49^ and peaks at 1150, 1120, and 1006 cm^–1^ belonged to Si–O–Si and Si–O–Al stretching vibration.^47^ Peaks at 870 and 800 cm^–1^ could be attributed to O–H stretching of FeOOH species, while the peak at 560 cm^–1^ is assigned to the Fe–O stretching mode.^42^

**Figure 6:**
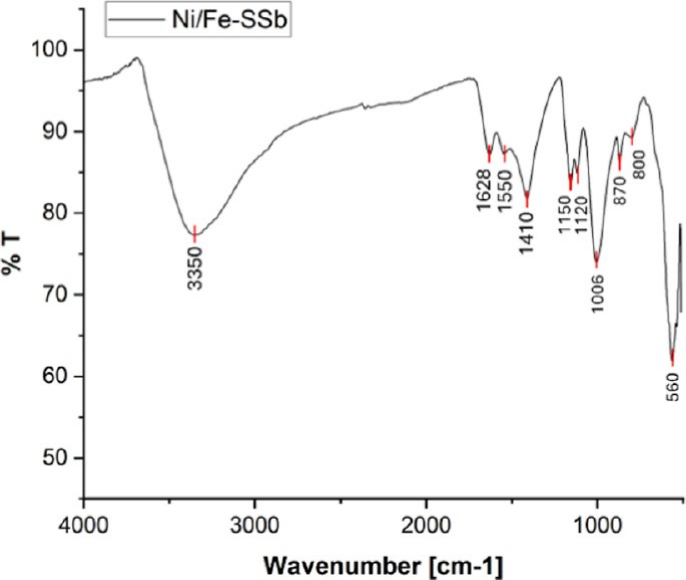
FT-IR spectrum of the catalyst **Ni/Fe-SSb**.

The FE-SEM image of **Ni/Fe-SSb** (Figure 7A,B) highlighted a micro- and nanostructured morphology of the material, and the EDX analysis (Figure 7) showed a homogeneous distribution of all elements and revealed the presence of chloride impurities.

**Figure 7:**
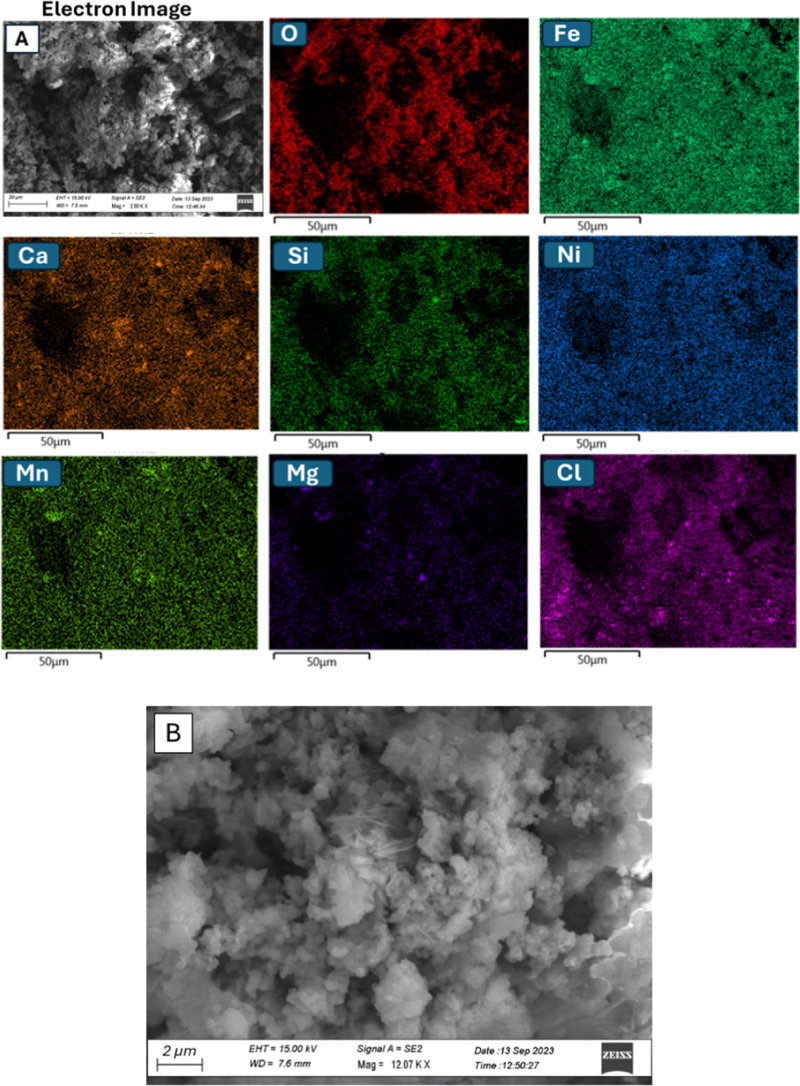
(A) FE-SEM image and corresponding EDX maps and (B) FE-SEM image at a different magnification of **Ni/Fe-SSb**.

The XRD diffractogram of **Ni/Fe-SSb** (Figure 8) showed the presence of multiple crystalline phases such as magnetite (Fe_3_O_4_), metallic nickel (Ni), anhydrite (CaSO_4_), calcite (CaCO_3_), and wüstite (FeO). The most intense reflections are attributed to magnetite, which justified the strong magnetic properties of the material and its black color. Rietveld analysis^32,33^ revealed that magnetite was deposited as nanoparticles with a mean size diameter of 32.5 ± 1 nm. Additional peaks corresponding to metallic nickel confirmed the successful incorporation of the active metal phase, similar to what was observed in **Ni/Fe-SS**. In the case of **Ni/Fe-SSb**, the mean size diameter of the Ni nanoparticles was 45.9 ± 1.2 nm, indicating again that potentially catalytically active nickel species formed during the calcination step of the material.^48^

**Figure 8:**
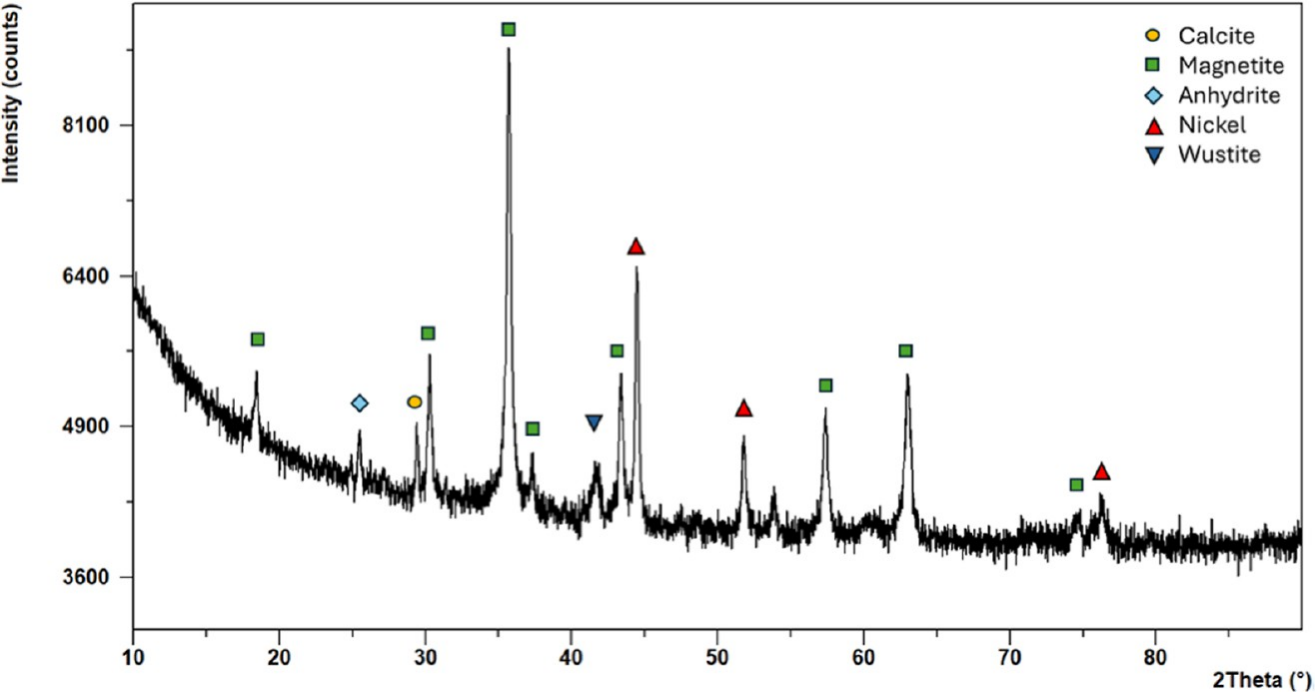
XRD diffractogram of **Ni/Fe-SSb**.

The TEM images of **Ni/Fe-SSb** (Figure 9) showed agglomerates of nanoparticles with irregular morphologies and poorly defined edges. The particle distribution appears to be nonuniform, with denser (darker) areas that could indicate a higher concentration of magnetite and metallic Ni or metallic agglomerates.

**Figure 9:**
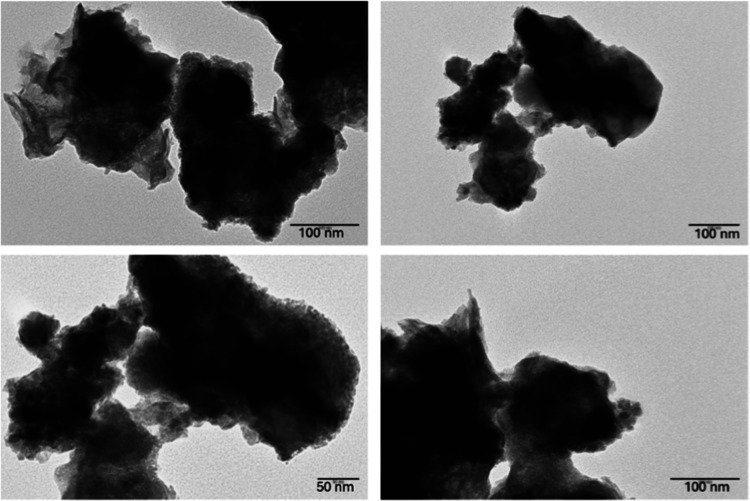
TEM images of **Ni/Fe-SSb** at different magnifications.

The analyses of **Ni/Fe-SS** and **Ni/Fe-SSb** indicate that the two different synthesis procedures led to the formation of different materials, both containing iron oxides and metallic nickel. In the absence of sodium hydroxide (Figure 1a), the red-brown solid obtained prior to calcination contained Ni­(II) species impregnated on the surface of **Fe-SS** after water evaporation. During calcination at 300 °C under dihydrogen, the Ni­(II) centers were reduced to metallic nickel. Several studies have reported the reduction of solid Ni­(OH)_2_, NiO, and even NiCl_2_ to nickel nanoparticles (NPs) under H_2_ at various temperatures.^50,51^ In any case, it has been observed that the reduction of Ni­(II) to Ni NPs under H_2_ is favored by increasing both the temperature and calcination time^52,53^ and by using a solid support that promotes nucleation.^54^ Furthermore, the presence of Fe­(II) has been reported as beneficial for the reduction of Ni­(II) to Ni(0), since Fe­(II) is oxidizable to Fe­(III).^55^

Comparison of **Ni/Fe-SS** and **Ni/Fe-SSb** shows that in both catalysts, nickel is present in the form of nanocrystalline Ni(0), whereas the iron oxides are in the form of nanohematite for **Ni/Fe-SS** and nanomagnetite for **Ni/Fe-SSb** (XRD analysis, Figures 4 and 8). Both Ni and iron oxides were uniformly distributed onto the surface of the catalysts, thus anticipating potential synergic effects of iron oxides and nickel in catalysis. In addition, **Ni/Fe-SS** contained both acidic and alkaline sites due to presence of Lewis acids, basic metal oxides, and amphiphilic AlO­(OH), while **Ni/Fe-SSb** contained only basic sites, as suggested by the presence of magnetite, whose formation occurs in alkaline environments.^56^

### 3.3 Catalytic Partial Hydrogenation of FAMEs

The WCO FAMEs mixture employed in the present work was constituted of methyl linoleate (C18:2, 41.9%), methyl oleate (C18:1, 48.9%), methyl stearate (C18:0, 3.2%), methyl palmitate (C16:0, 5.7%), and methyl arachidate (C20:0, 0.3%) (Figure 10). Its main biodiesel parameters were unsatisfactory, being its oxidation stability (OS) and its iodine value (IV) equal to 5.4 h and 121 g/100 g, respectively, thus unfitting the European thresholds (OS > 8 h; IV < 120 g/100 g).^57^ On the other hand, the cold filter plugging point (CFPP) value (−8.5 °C) of the WCO FAMEs mixture was pretty good because it was far behind the EU limit (<10 °C). These features reflected the composition of the mixture, which contained a high amount of polyunsaturated methyl ester (C18:2) that caused the poor values of both OS and IV due to its chemical instability (tendency to polymerize, oxidize, and go rancidity) and the good fluidity properties expressed by CFPP. To improve OS and IV parameters, it was necessary to convert all C18:2 (linoleate) into C18:1 ester (oleate) by catalytic partial hydrogenation, avoiding the formation of C18:0 (stearate, Figure 11). In fact, methyl stearate is solid at RT, and a high amount of C18:0 negatively affects the fluidity parameters and CFPP value of the biodiesel. In the present work, only parameters related to C18:2, C18:1, and C18:0 relative composition were considered, being the molar amount of C16:0 and C20:0 unaffected by the partial hydrogenation.

**Figure 10:**
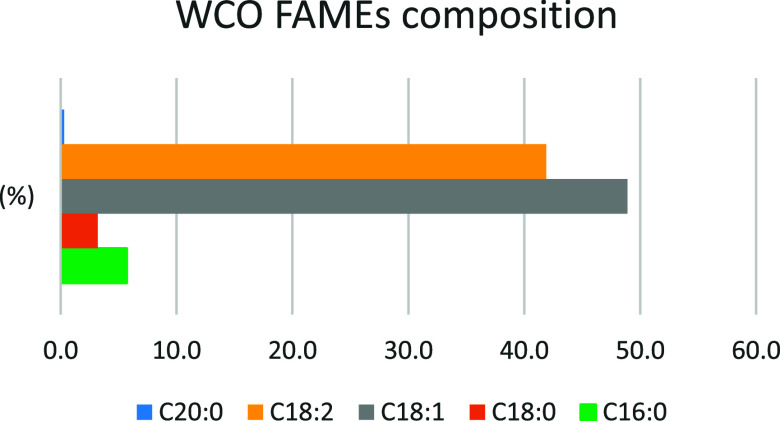
FAMEs molar composition of the WCO used in the present study.

**Figure 11:**
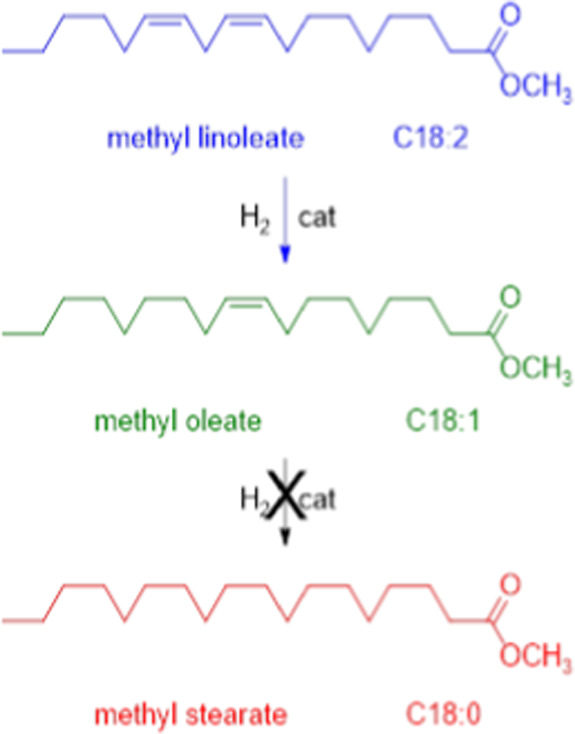
Catalytic partial hydrogenation of methyl linoleate in WCO biodiesel.

**Ni/Fe-SS** and **Ni/Fe-SSb** were employed as the catalysts for the partial hydrogenation of the WCO biodiesel. Catalytic tests for the optimization of reaction conditions were carried out by using **Ni/Fe-SS** as the catalyst.

The partial hydrogenation reaction was carried out by using molecular hydrogen or sodium borohydride (CTH method) as the reductant.

#### 3.3.1 Catalytic Tests: Optimization of the FAMEs Upgrading Conditions under Dihydrogen

Preliminary tests were carried out aiming at optimizing reaction conditions, by using in any case fixed amount of **Ni/Fe-SS** (50 mg) and WCO biodiesel (125 mg) in methanol (5.0 mL), and varying parameters, such as temperature, reaction time, and hydrogen pressure. The relevant results are summarized in Table 4, which reports the relative molar percentage amounts of the C18 methyl esters in the hydrogenated FAMEs mixture together with the main parameter values affected by their content. The current biodiesel parameters of the obtained biodiesel mixtures were calculated according to equations reported in the study of Talebi et al.^58^ who developed a user-friendly software called BiodieselAnalyzer designed to predict biodiesel parameters using the fatty acid methyl ester profile as the only input required.

**Table 4:** Catalytic Tests in Biodiesel Hydrogenation Reaction under Dihydrogen^a^

entry	catalyst	*T* (°C)	*t* (h)	*P* (bar)	C18:0^b^(%)	C18:2^b^(%)	C18:1^b^(%)	CFPP^c^ (°C)	OS^c^ (h)	IV^c^ (g/100 g)
1					3.8	44.4	51.8	–8.5	5.4	121
2	**Ni/Fe-SS**	100	15	10	7.0	11.9	81.1	–4.2	13.0	89.7
3	**Ni/Fe-SS**	100	10	10	6.3	15.5	78.2	–5.4	10.6	93.2
4	**Ni/Fe-SS**	100	10	5	6.2	15.9	77.9	–5.3	10.5	88.2
5	**Ni/Fe-SS**	100	6	5	6.1	16.0	77.9	–5.1	10.2	87.6
6	**Ni/Fe-SS**	70	6	5	6.0	15.9	78.1	–5.4	10.3	88.1
7	**Ni/Fe-SS**	100	10	2.5	6.0	26.1	67.9	–5.7	7.4	101.9
8	**Ni/Fe**-S**Sb**	70	6	5	7.7	11.1	81.2	–3.3	13.8	87.6
9	**Fe-SS**	100	10	10	4.4	31.8	63.8	–8.5	6.4	111.2
10	**Ni-SS**	100	10	10	8.2	25.8	66.0	–2.56	7.4	95.2
11	**Ni/Fe-SS** d	70	12	5	12.0	6.5	81.5	3.2	21.9	79.8

a Reaction conditions: 50 mg of catalyst, 125 mg of biodiesel, and 5.0 mL of methanol.

b Values reported represent the relative molar percentages of C18 esters (C18:1, C18:2, and C18:0).

c Key parameters biodiesel values according to REGULATION (EU) 2022/2383; the threshold established by European standards are CFPP < 10 °C; OS > 8 h; and IV < 120 g/100 g. Values in red are outside these limits.

d Reaction conditions: 500 mg of catalyst, 1.25 g of biodiesel, and 10.0 mL of methanol.

Entry 1 (Table 4) reports the relative molar percentage of C18 chains in starting WCO FAMEs, with unsatisfactory values of OS and IV. By carrying out the hydrogenation reaction at 100 °C under 10 bar of H_2_ for 15 h (entry 2), all parameters fell within the limits set by EU regulation since the C18:1 molar percentage increased to 81.1%, C18:2 decreased to 11.9%, and C18:0 raised to only 7.0% with respect to the starting biodiesel (entry 1). Decreasing the reaction time to 10 h (entry 3) or both reaction time and hydrogen pressure (10 h and 5 bar of H_2_, entry 4) resulted in slightly higher amounts of C18:2, slightly lower amounts of C18:1, and good OS and IV parameters. Similar good results were obtained by decreasing the reaction time to 6 h, keeping the H_2_ pressure at 5 bar and the temperature at 100 °C (entry 5) and subsequently decreasing the temperature to 70 °C (entry 6). Only when the hydrogen pressure was dropped to 2.5 bar (entry 7), the hydrogenation of C18:2 to C18:1 occurred to an insufficient extent (67.9% of C18:1), resulting in an unacceptable OS (7.4 h).

The optimal reaction conditions used in entry 6 were employed for testing the catalytic activity of **Ni/Fe-SSb**, which gave very good results in terms of CFPP, OS, and IV parameters (entry 8, Table 4).

Regarding temperature parameter, it is known that an increase in temperature promotes the isomerization of *cis*-monounsaturated FAMEs into the thermodynamically favored^59^ *trans* isomers.^13,60,61^ In addition, it has been observed that high temperatures increase the conversion to fully saturated FAMEs^62–64^ and increase the reaction rate.^63^ To achieve an optimal compromise between reaction speed and selectivity toward *cis*-monounsaturated FAMEs, the recommended temperature range varies between 70 and 120 °C,^62–64^ as found for **Ni/Fe-SS** and **Ni/Fe-SSb** catalytic systems (optimal temperature = 70 °C). Indeed, this finding is in line with what has been observed for some Ni-based bimetallic systems for which the optimal temperature has been set at 100 °C.^65^ Another significant parameter is the pressure, which influences the final products of hydrogenation. Numwong et al.^61^ showed that an increase in pressure promotes the conversion of polyunsaturated FAMEs, but it also increases the formation of *trans*-monounsaturated FAMEs. Similar results were reported by Phumpradit et al.,^60^ who found an increase in both *trans*-monounsaturated isomers and fully hydrogenated FAMEs with increasing pressure. Zhu et al.,^63^ on the other hand, observed that higher pressure mainly leads to a higher reaction rate. Generally, the optimal hydrogen pressure does not exceed 5 bar, as reported for both monometallic^60,61,63^ and bimetallic systems.^65^ By using **Ni/Fe-SS** as the catalyst, *cis* to *trans* isomerization of C18:1 ester was observed in small extent. In fact, under the optimized reaction conditions (entry 6, Table 4), the *cis*/*trans* C18:1 molar ratio in the hydrogenated blend was equal to 8, while in the case of the **Ni/Fe-SSb** catalytic system, the C18:1 *cis*/*trans* molar ratio was approximate to 4 (entry 8, Table 4). Indeed, the *cis* to *trans* isomerization should be avoided^16^ since the *trans* isomer solidifies at higher temperature than the *cis* one, even if it has been claimed that a small amount of *trans* C18:1 in biodiesel is advantageous due to its good lubricant properties.^66^

To verify the possible synergistic effect between iron oxide and nickel in **Ni/Fe-SS** and to gain insight into the individual contributions of each component within the system studied, the catalytic activity of SS supporting only Fe (**Fe-SS**) and SS supporting only Ni (**Ni-SS**), obtained by precipitation of Ni­(OH)_2_ onto SS followed by calcination under 5 bar of H_2_ at (300 °C), was tested.

When dihydrogen was used as a reducing agent, neither **Fe-SS** nor **Ni-SS** produced biodiesel mixtures with adequate oxidative stability, being the values obtained below the threshold established by European standards (entries 9–10, Table 4). These results were observed despite the use of more stringent experimental conditions—such as higher temperature, prolonged reaction time, and higher hydrogen pressure than the optimized ones used with **Ni/Fe-SS** and **Ni/Fe-SSb** catalysts (entries 6 and 8), confirming a beneficial synergistic effect between iron oxides and nickel in the hydrogenation reaction.^23,26,27^ Therefore, hydrogenation experiments using the **Ni/Fe-SS** catalyst revealed that optimal reaction conditions were achieved at 70 °C, under 5 bar of H_2_ for 6 h (entry 6, Table 4). Under these conditions, among C18 methyl esters, the C18:1 content rose to 78.1%, while C18:2 was reduced to 15.9%, and only 6.0% of C18:0 was formed. This selective enrichment in monounsaturated chains significantly improved the fuel quality, resulting in an oxidative stability of 10.3 h and an IV of 88.1 g I_2_/100 g, thus fully meeting EU standards (OS ≥ 8 h, IV ≤ 120). Compared to the initial biodiesel (entry 1, Table 4), these results represent a 90% improvement in oxidative stability and a 27% reduction in IV. When the same optimized conditions were applied using the **Ni/Fe-SSb** catalyst, an even better performance was observed (entry 8, Table 4). The final FAME mixture showed 81.2% C18:1, 11.1% C18:2, and 7.7% C18:0, achieving an OS of 13.8 h and an IV of 87.6 g of I_2_/100 g. Compared to the starting biodiesel (entry 1, Table 4), this corresponds to a 155.6% increase in OS and a 27.6% decrease in IV. Overall, both catalysts demonstrated high selectivity toward C18:1 formation and were effective in converting polyunsaturated esters to the monounsaturated ones, while minimizing excessive saturation.

For practical relevance and potential applications, it is essential to include data from scale-up experiments in the gram range. For this reason, a scale-up test in gram scale was carried out by using **Ni/Fe-SS** as the catalyst under H_2_, obtaining satisfactory results (entry 11, Table 4).

#### 3.3.2 Catalytic Tests: Optimization of the FAMEs Upgrading Conditions with NaBH_4_

NaBH_4_ is a well-known safe source of dihydrogen when reacts with methanol in the presence of a metal catalyst (eq 1).^67^

1 $$NaBH_{4}+CH_{3}OH\overset{cat}{\to }NaB{\left(OCH_{3}\right)}_{4}+4H_{2}$$

Partial hydrogenation tests of WCO FAMEs were carried out also using NaBH_4_ as the reductant. The relevant results are reported in Table 5.

**Table 5:** Catalytic Tests with **Ni/Fe-SS** in Biodiesel Hydrogenation Reaction Using NaBH_4_ ^a^

entry	catalyst	*t* (min)	NaBH_4_ (mmol)	C18:0^b^(%)	C18:2^b^(%)	C18:1^b^(%)	CFPP^c^ (°C)	OS^c^ (h)	IV^c^ (g/100 g)
1	**Ni/Fe-SS**	30	5	>99	0	0			
2	**Ni/Fe-SS**	20	1.3	21.9	0.4	77.7	17.6	318.8	64.3
3	**Ni/Fe-SS**	20	0.8	16.0	2.1	81.9	9.1	63.8	71.2
4	**Ni/Fe-SS**	20	0.5	13.0	2.3	84.7	4.5	57.6	76.2
5	**Ni/Fe-SS**	20	0.26	9.7	11.0	79.3	–0.24	14.0	85.7
6	**Ni/Fe-SS**	40	0.26	9.9	10.7	79.4	0.4	14.7	82.8
7	**Ni/Fe**-**SSb**	20	0.26	13.4	17.0	69.6	5.6	10.2	84.8
8	**Fe-SS**	20	0.26	5.0	46.0	49.0	–6.6	5.2	116.4
9	**Ni-SS**	20	0.8	16.7	22.8	60.5	10.2	8.2	88.1

a Reaction conditions: 50 mg of catalyst, 125 mg of biodiesel, 5.0 mL of methanol, and RT.

b Values reported represent the relative molar percentages of C18 esters (C18:1, C18:2, and C18:0).

c Key parameters biodiesel values according to REGULATION (EU) 2022/2383; the threshold established by European standards is CFPP <10 °C; OS > 8 h; and IV < 120 g/100 g. Values in red are outside these limits.

A preliminary test was carried out at RT in the presence of 5.0 mmol of NaBH_4_ for 30 min, resulting in total hydrogenation of all C18 carbon–carbon double bonds, and yielding a waxy solid unsuitable for biodiesel purposes (entry 1, Table 5). For this reason, in the subsequent catalytic tests, the temperature was kept at RT, and the amount of NaBH_4_ and the reaction time were decreased. By using 1.3 mmol of NaBH_4_ and 20 min of reaction time (entry 2, Table 5), the C18:2 component almost disappeared, but the methyl stearate molar percentage increased too much (21.9% of the total C18 esters sum), leading to an unsatisfactory value of CFPP (17.6 °C). Better results were observed by further decreasing the amount of the employed NaBH_4_ to 0.80 mmol 0.50 and 0.26 mmol (entries 3, 4, and 5, respectively, Table 5). On the basis of the obtained results, the best catalytic parameters resulted the following: *t* = 20 min, 0.26 mmol of NaBH_4_, and RT (entry 5, Table 5). Thus, the **Ni/Fe-SS** catalyst showed a high degree of selectivity toward monounsaturated chains: among C18 esters, C18:1 reached 79.3%, while C18:2 was reduced to 11.0%, and C18:0 increased moderately to 9.7%. These values translated into excellent fuel properties: the oxidative stability improved to 14.0 h, and the IV dropped to 85.7 g of I_2_/100 g, both well within the EU regulatory limits. Compared to the initial biodiesel (entry 1, Table 4), this corresponds to a 159.3% increase in the OS and a 29.2% reduction in IV.

To check the residual potential activity of the catalytic system, the reaction time was prolonged to 40 min (entry 6, Table 5), but no substantial variation of the composition of the FAMEs mixture was observed. The optimal reaction conditions were used for testing the catalytic activity of **Ni/Fe-SSb** (entry 7, Table 5), which resulted less selective toward the target C18:1 product than **Ni/Fe-SS,** being the amount of methyl stearate and methyl linoleate 13.4 and 17.0%, respectively (entry 7, Table 5), higher than those obtained with **Ni/Fe-SS** (9.7 and 11.0%, respectively, entry 5, Table 5), however still compliant with the EU standards (OS = 10.2 h and IV = 84.8 g I_2_/100 g), reaching an improvement of 89% and 30% for OS and IV, respectively, with respect to the starting FAME mixture (entry 1, Table 4).

To confirm the synergistic effect between iron oxides and Ni catalyst, **Fe-SS** and **Ni-SS** were tested in the partial hydrogenation of FAMEs using NaBH_4_ as the reducing agent (entries 8–9, Table 5). **Fe-SS** showed no catalytic activity (entry 8, Table 5), while **Ni-SS** exhibited a moderate catalytic activity (entry 9, Table 5) sensibly lower with respect to the **Ni/Fe-SS** and **Ni/Fe-SSb** systems.

### 3.4 Recyclability Tests

Recyclability studies were conducted on **Ni/Fe-SS** and **Ni/Fe-SSb** by using both dihydrogen and sodium borohydride as reducing agents, under the optimized reaction conditions reported in Tables 4 and 5.

By using dihydrogen as the reducing agent, **Ni/Fe-SS** recovered after the first run (entry 2, Table 6) and resulted active also in the second cycle (entry 3, Table 6). Starting from the third cycle (entry 3, Table S1), too high a concentration of methyl linoleate in the hydrogenated mixture was detected, leading to an unsatisfactory decrease of oxidative stability (below 8 h). The catalytic activity of **Ni/Fe-SS** decreased with the recycles (entries 4 and 5, Table S1), and the FAMEs mixture recovered after the fifth cycle (entry 5, Table S1) was similar in composition to the starting biodiesel blend (entry 1, Table 6). Notably, the concentration of stearate remained constant throughout the cycles.

**Table 6:** Recyclability Tests Using H_2_ or NaBH_4_ as the Reducing Agent and **Ni/Fe-SS** or **Ni/Fe-SSb** as the Catalyst^a^

entry	catalyst	reductant	*T* (°C)	*t* (min)	C18:0^b^(%)	C18:2^b^(%)	C18:1^b^(%)	CFPP^c^ (°C)	OS^c^ (h)	IV^c^ (g/100 g)
1					3.8	44.4	51.8	–8.5	5.4	121
2	**Ni/Fe-SS**	H_2_ (5 bar)	70	360	6.3	15.3	78.4	–5.4	10.7	93.0
3^d^	“	“	“	“	7.7	11.1	81.2	–3.3	13.8	87.6
4	“	NaBH_4_ (0.26 mmol)	25	20	9.7	11.0	79.3	–0.24	14.0	85.7
5^d^	“	“	“	“	12.6	15.5	71.9	4.3	10.9	84.4
6	Ni/Fe-SSb	H_2_ (5 bar)	70	360	7.7	11.1	81.2	–3.3	13.8	87.6
7^d^		“	“	“	3.9	43.0	53.1	–8.5	5.6	116.3
8		NaBH_4_ (0.26 mmol)	25	20	13.5	16.9	69.6	5.6	10.2	84.8
9^d^		“	“	“	10.2	36.6	53.2	1.0	6.1	103.5
10^e^	**Ni/Fe-SS**	H_2_ (5 bar)	70	360	7.6	22.7	69.7	–2.35	8.5	88.4
11^f^	**Ni/Fe**-**SSb**	H_2_ (5 bar)	70	360	10.8	19.4	69.8	1.82	9.3	84.8

a Reaction conditions: 100 mg of catalyst and 250 mg of biodiesel in 10.0 mL of methanol (the other parameters are reported in Table 6).

b Values reported represent the relative molar percentages of C18 esters (C18:1, C18:2, and C18:0).

c Key parameters biodiesel values according to REGULATION (EU) 2022/2383; the threshold established by European standards are CFPP <10 °C; OS > 8 h; and IV < 120 g/100 g. Values in red are outside these limits.

d Recycle of the previous run.

e The catalyst was used for five subsequent cycles, then it was reactivated by calcination under H_2_ (initial pressure = 5 bar) at 300 °C for 30 min.

f The catalyst was used for three subsequent cycles, and then it was reactivated by calcination under H_2_ (initial pressure = 5 bar) at 300 °C for 30 min.

A loss of activity with recycling was also observed by using sodium borohydride for **Ni/Fe-SS** as the reducing agent, where the limits for the three biodiesel parameters were maintained up to the third cycle (entries 4–5, Table 6; entry 3, Table S2). Passing from the fourth to fifth cycle, the relative amounts of FAMEs remained relatively unchanged, with only the oxidative stability parameter falling slightly below the regulatory threshold (entries 4 and 5, Table S2).

The recyclability tests carried out on the **Ni/Fe-SSb** magnetic catalyst both under dihydrogen and with NaBH_4_ revealed unsatisfactory results in the recycles (entries 7 and 9, Table 6), becoming totally inactive in the third run (entry 3, Tables S3 and S4).

However, calcination of spent **Ni/Fe-SS** and **Ni/Fe-SSb** at 300 °C for 30 min under 5 bar of dihydrogen allowed to reactivate the catalysts, which regained their initial catalytic activity in the partial hydrogenation of FAMEs under H_2_ (entries 10–11, Table 6).

## 4 Discussion

Both **Ni/Fe-SS** and **Ni/Fe-SSb** displayed high catalytic activity and selectivity in the first reaction run both under dihydrogen and in the presence ofNaBH_4_, with the **Ni/Fe-SS** catalyst being active in the first two recycles and the **Ni/Fe-SSb** catalyst being inactive after the first cycle, despite a very similar elemental composition. XRF analysis revealed that no loss of Ni or Fe occurred during duty for both **Ni/Fe-SS** and **Ni/Fe-SSb**, suggesting that the drop of activity registered with cycles should be rather ascribed to the formation of the inactive NiO layer during workup.^16^ In fact, the observed reactivation of spent **Ni/Fe-SS** and **Ni/Fe-SSb** (entries 10-11, Table 6) by means of calcination under H_2_ at 300 °C indicated a plausible reduction of the inert NiO layer to the catalytically active Ni nanoparticles during treatment.

However, the formation of NiO in the employed catalysts was not detected by XRD analyses (Figures S7 and S9) due to overlapping of diffraction peaks belonging to different species (anhydrite and magnetite signals superimposing to nickel oxide peaks). Moreover, XRF analyses confirmed that the total amount of nickel remained almost unchanged during recycles in both catalysts.

The difference in the recyclability between **Ni/Fe-SS** and **Ni/Fe-SSb** is more difficult to rationalize. The main difference between the two catalysts lies in the nature of the iron oxide nanoparticles covering the SS support, namely, hematite (α-Fe_2_O_3_) in **Ni/Fe-SS** and magnetite (Fe_3_O_4_) in **Ni/Fe-SSb**. This indicates a higher basicity of the support in the case of **Ni/Fe-SSb** with respect to **Ni/Fe-SS**, given that the formation of magnetite from a Fe­(II)/Fe­(III) system occurs under alkaline and anaerobic conditions,^68^ whereas less alkaline conditions give rise to the formation of α-Fe_2_O_3_.^69^

The fact that the support of **Ni/Fe-SSb** is more alkaline than in **Ni/Fe-SS** was established by carrying out a Hammett test^70^ on both materials, following the same procedure employed in a previous study.^34^ In fact, the *H*_0_ of **Ni/Fe-SS** was ca. 4.8, while the *H*_0_ of **Ni/Fe-SSb** resulted higher than 6.0 (and lower than 8.2, see the Supporting Information, Figure S4). Although no conclusive statement can be drawn based on the available data, the different basicity of the supports may be taken as responsible for the different recyclability observed for **Ni/Fe-SS** and **Ni/Fe-SSb**. In fact, Hammett tests carried out on the spent **Ni/Fe-SS** catalyst recovered after five reaction cycles showed an 8.2 > *H*_0_ > 6.0 (thus higher basicity compared to the *H*_0_ ≈ 4.8 of the fresh **Ni/Fe-SS** catalyst, Figure S5).

In all catalytic tests, **Ni/Fe-SS** and **Ni/Fe-SSb** systems were selective toward the formation of C18:1 ester, avoiding the total hydrogenation. Indeed, as reported in the literature,^16,71,72^ the hydrogenation of C18:2 methyl ester is faster than the hydrogenation of the C18:1 ones due to formation during the catalytic pathway of conjugate dienes easier to be hydrogenated owing to generation of allylic intermediates. The production of conjugate dienes (due to migration of the C=C bond) as well as the *cis* to *trans* isomerization of unsaturated methyl esters could be explained on the basis of the generally accepted hydrogenation mechanism^62^ ,^72^ depicted in Figure 12 for the **Ni/Fe-SS** and **Ni/Fe-SSb** catalytic systems. After oxidative addition of H_2_ to the Ni nanoparticles (NPs) and coordination of the π double bond of the olefin, the subsequent hydrogen migration to the carbon atom (step a, Figure 12) is reversible; thus, the inverse reformation of the C=C double bond could generate the thermodynamically favored *trans* isomer (step c, Figure 12) or could give double bond migration (step d, Figure 12), prior to yielding the hydrogenated product (step b, Figure 12).

**Figure 12:**
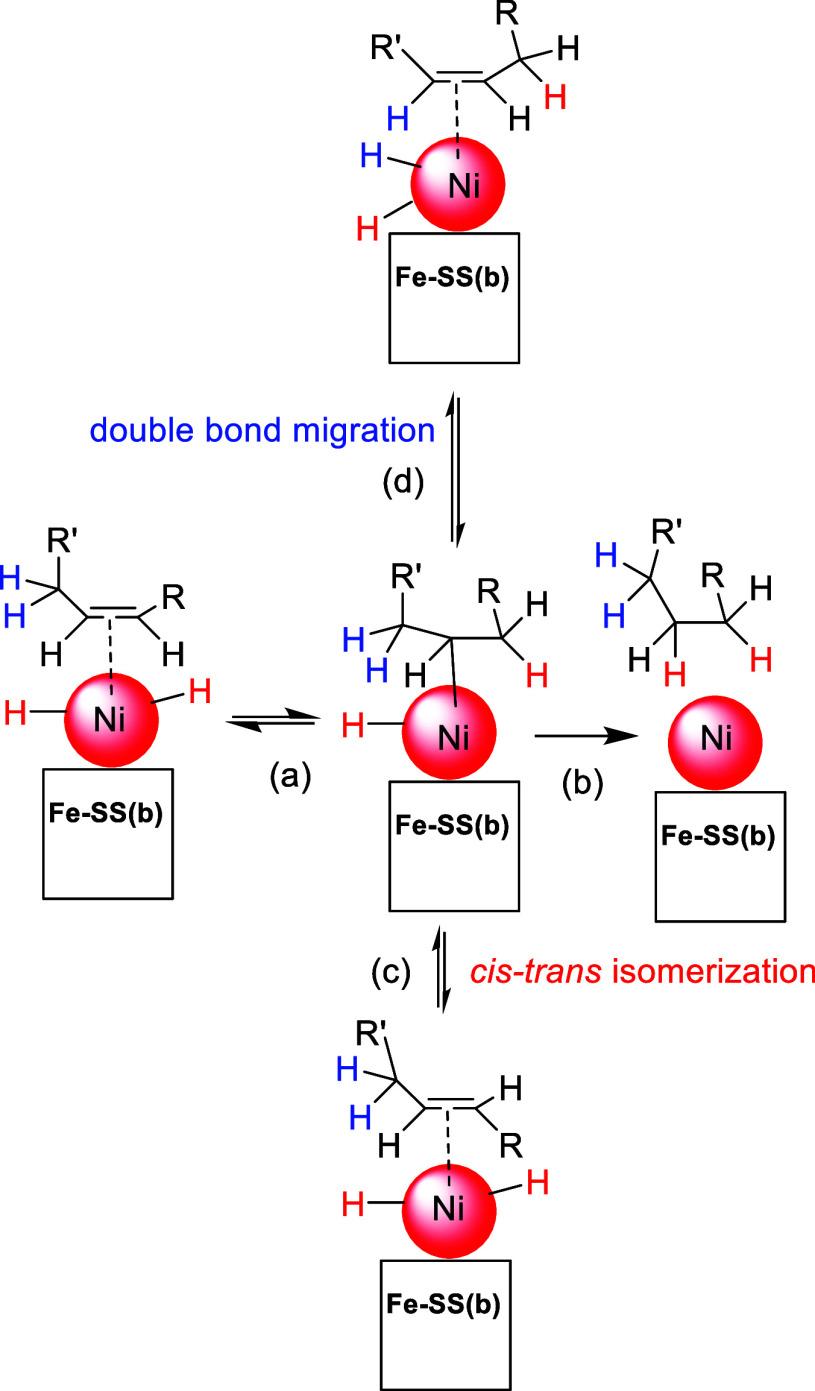
Proposed mechanism of hydrogenation of FAMEs catalyzed by **Ni/Fe-SS** or **Ni/Fe-SSb**.

Indeed, the different acidity properties of the surface in **Ni/Fe-SS** and **Ni/Fe-SSb** could affect the mechanism pathway of the dihydrogen dissociation. In fact, it has worldwide recognized that basic sites on the catalyst support can promote the heterolytic cleavage of H_2_;^73^ thus, in the **Ni/Fe-SSb** catalytic systems, the homolytic and the heterolytic splitting of dihydrogen can occur simultaneously. On the contrary, by using **Ni/Fe-SS**, only the homolytic dissociation of H_2_ should arise. This phenomenon might affect the selectivity toward the *cis* to *trans* isomerization (step c, Figure 12) and the double bond migration (step d, Figure 12) since **Ni/Fe-SSb** resulted more prone to give isomerization (C18:1 *cis*/*trans* molar ratio = 4 in the first run) compared to **Ni/Fe-SS** (C18:1 *cis*/*trans* molar ratio = 8 in the first run).

## 5 Conclusions

Treating steel slags with iron and nickel salts produced new Ni/FeO_*x*_-supported catalysts containing nickel/hematite nanoparticles (**Ni/Fe-SS)** or nickel/magnetite nanoparticles (**Ni/Fe-SSb)**. The new materials were tested in the partial hydrogenation reaction of biodiesel obtained from WCO, showing high activity and selectivity toward the C18:1 methyl ester in the first run either under H_2_ or by using NaBH_4_ as the reductant, thus yielding hydrogenated fatty acid methyl ester blend fitting the European regulation on cold flow and OS parameters. Both **Ni/Fe-SS** and **Ni/Fe-SSb** catalysts showed a decrease in their catalytic activity with the recycles, more evident for **Ni/Fe-SSb** with respect to **Ni/Fe-SS**, presumably due to the formation of inactive NiO under aerobic workup procedures. However, the spent catalysts could be reactivated by calcination under dihydrogen, which is suitable to reduce the inactive nickel oxide to the catalytically active Ni nanoparticles. This work is an example of waste valorization where biodiesel coming from WCO is upgraded by means of novel bimetallic catalysts synthesized from steel slags following circular economy principles.

## Supplementary Material



## Data Availability

The data supporting the findings of this study are included in this article and its Supporting Information.

## Notes

Experimental part (synthesis of **Ni-SS**; Hammett test on **Ni/Fe-SS** and **Ni/Fe-SSb**); FT-IR spectrum of the **Fe-SS** support; FE-SEM image and EDX maps of the **Fe-SS** support; XRD diffractogram of the **Fe-SS** support; Hammett test results for **Ni/Fe-SS**, **Ni/Fe-SSb**, and **SS** using phenolphthalein as an indicator, with a methyl red and bromocresol blue mixture; Hammett test results on the **Ni/Fe-SS** catalyst after the first and fifth reaction cycles using a methyl red and bromocresol blue mixture; XRD diffractogram of **Ni/Fe-SS** before use in catalysis; XRD diffractogram of **Ni/Fe-SS** after use in catalysis; XRD diffractogram of **Ni/Fe-SSb** before use in catalysis; XRD diffractogram of **Ni/Fe-SSb** after use in catalysis; recyclability tests using dihydrogen as the reducing agent and **Ni/Fe-SS** as the catalyst; recyclability tests using NaBH_4_ as the reducing agent and **Ni/Fe-SS** as the catalyst; recyclability tests using dihydrogen as the reducing agent and **Ni/Fe-SSb** as the catalyst; and recyclability tests using NaBH_4_ as the reducing agent and **Ni/Fe-SSb** as the catalyst (PDF)
