# Supplementary material for: Partial Hydrogenation of Waste Cooking Oil Biodiesel Catalyzed by Iron Oxide/Nickel Nanoparticles Supported on Steel Slags
Source: ACS Omega. 2025 Nov 5;10(45):54070–85. doi: 10.1021/acsomega.5c05619 (PMC12631674; doi:10.1021/acsomega.5c05619)
Supplement: Supplementary file 1 [file ao5c05619_si_001.pdf]

Partial hydrogenation of waste cooking oil biodiesel catalysed by iron oxide/nickel nanoparticles supported on steel slags

Maria Stella Leone,[a] Piero Mastrorilli,[a] Ernesto Mesto[b], Emanuela Schingaro[b], Roberto Comparelli[c], Carlo Porfido[d], Maria Michela Dell'Anna[a*]

[a] Department of Civil, Environmental, Land, Building Engineering and Chemistry, Politecnico di Bari, 4 via Orabona, Bari, 70125, Italy
[b] Department of Geomineralogy, University of Bari, 4 via Orabona, Bari, I-70125, Italy
[c] National Research Council, Institute for Physical and Chemical Processes - IPCF, 4 via Orabona, Bari, I-70125, Italy
[d] Department of Soil, Plant and Food Sciences, University of Bari, 165/A Via Amendola, Bari, 70126, Italy
* Email: mariamichela.dellanna@poliba.it

CONTENT

Experimental part……………………………………………………………………pag. S2

Figure S1 – FT-IR spectrum of the **Fe-SS** support……………………………………..pag. S3

Figure S2 – FE-SEM image (A) and EDX maps of **Fe-SS** support……………………pag. S4

Figure S3 - XRD diffractogram of the **Fe-SS** support…………………………………..pag. S5

Figure S4 - Hammett test results for **Ni/Fe-SS**, **Ni/Fe-SSb**, and **SS** (a) using phenolphthalein as an indicator, (b) with a methyl red and bromocresol blue mixture…………………………pag. S6

Figure S5 - Hammett test results on the **Ni/Fe-SS** catalyst after the first and fifth reaction cycles using a methyl red and bromocresol blue mixture……………………………………………pag.S7

Figure S6 - XRD diffractogram of the **Ni/Fe-SS** before use in catalysis……………………pag. S8

Figure S7 - XRD diffractogram of the **Ni/Fe-SS** after use in catalysis………………………pag. S9

Figure S8 - XRD diffractogram of the **Ni/Fe-SSb** before use in catalysis…………………pag. S10

Figure S9 - XRD diffractogram of the **Ni/Fe-SSb** after use in catalysis……………………..pag.S11

Table S1 - Recyclability tests using dihydrogen as the reducing agent and **Ni/Fe-SS** as catalyst……………………………………………………………………………………pag.S12

Table S2 - Recyclability tests using $NaBH_4$ as the reducing agent and **Ni/Fe-SS** as catalyst..pag.S13

Table S3 - Recyclability tests using dihydrogen as the reducing agent and **Ni/Fe-SSb** as catalyst……………………………………………………………………………………pag. S14

Table S4 - Recyclability tests using $NaBH_4$ as the reducing agent and **Ni/Fe-SSb** as catalyst..pag. S15

Experimental part

Synthesis of **Ni-SS**

$NiCl_2$□$6H_2O$ (3.00 mmol, 0.713 g) was dissolved in deionized water (40 mL), obtaining a green solution, which was added of SS (0.82 g). The resulting mixture (pH=12) was left under stirring at room temperature for 6 hours and dried overnight at 85°C. The resulting solid (1.3369 g) was calcined at 300°C for 30 minutes under an initial dihydrogen pressure of 5 bar, yielding a magnetic dark black solid material (0.9632 g), referred as **Ni-SS**.

*Hammett test on **Ni/Fe-SS** and **Ni/Fe-SSb***

Hammett tests were carried out on **Ni/Fe-SS** and **Ni/Fe-SSb** using a 10 mg/mL solution of phenolphthalein in methanol (pK_a = 9.5; color change: colorless-pink; pH range 8.2-10) and a mixture of methyl red (pK_a = 5.0; color change: red-yellow; pH range 4.8-6.0) and bromocresol blue (pK_a = 4.9; color change: yellow-blue; pH range 3.8-5.4) as indicators. Three drops of indicator (phenolphthalein or methyl red plus bromocresol blue) were put over 10 mg of catalyst lying on a glass plate. The test with phenolphthalein gave colorless solution (H_0 lower than 8.2) for both catalysts, while the test with methyl red plus bromocresol blue gave a green color for **Ni/Fe-SSb** (H_0 > 6.0, Figure S4) and an orange color for **Ni/Fe-SS** ($H_0 \approx 4.8$, Figure S4). For comparison, the Hammett test with phenolphthalein or methyl red plus bromocresol blue gave pink and green colors, respectively (H_0 > 10.0, Figure S4).

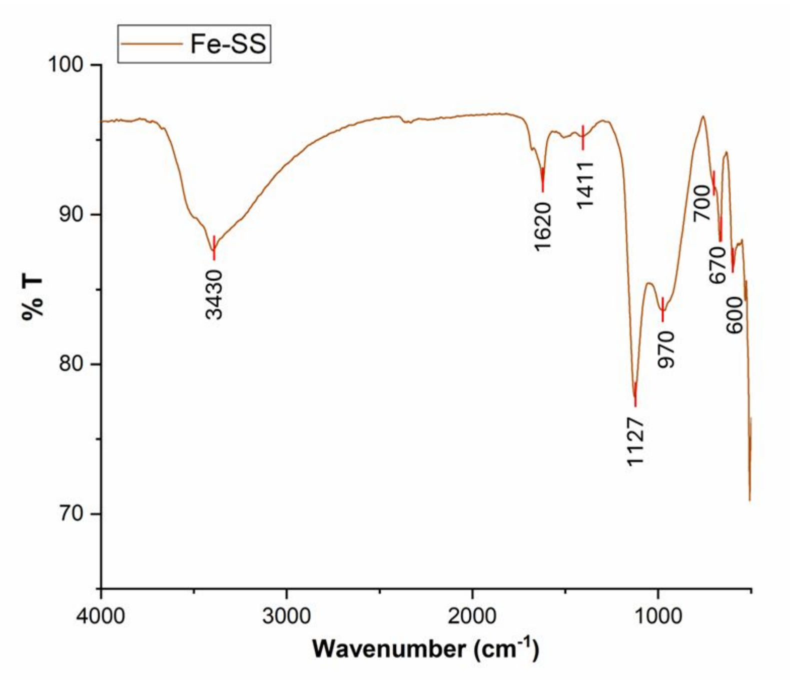

Figure S1 – FT-IR spectrum of the **Fe-SS** support

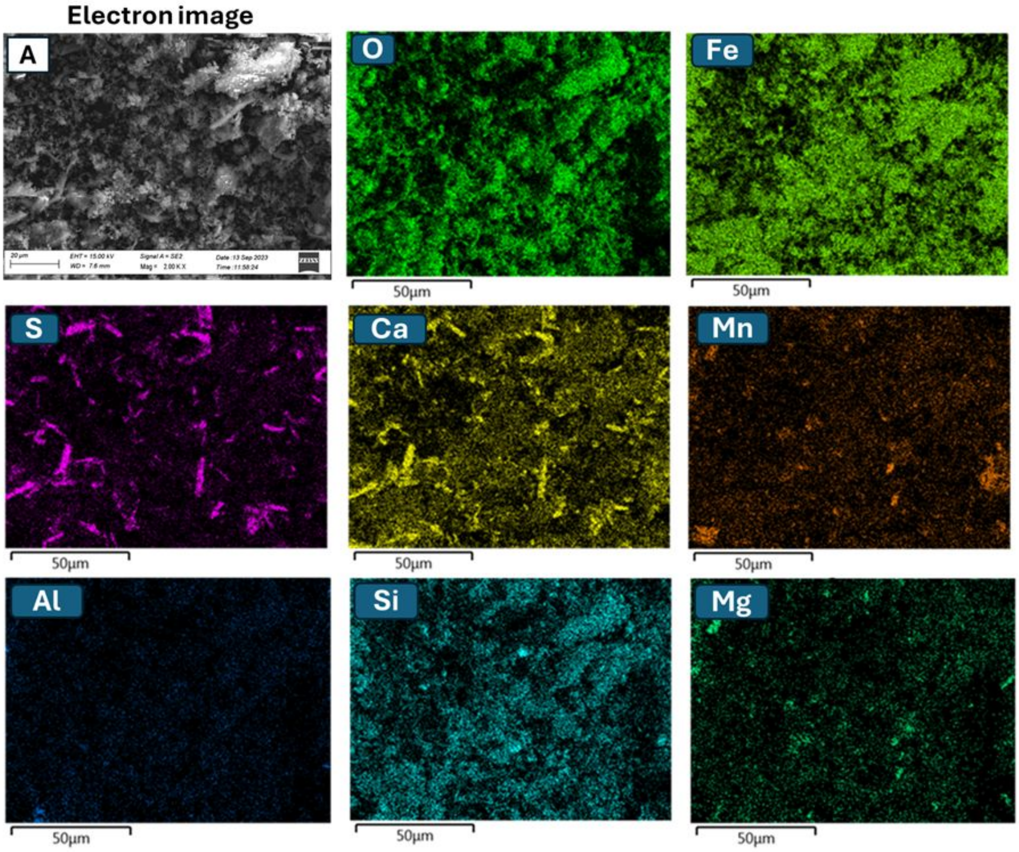

Figure S2 – FE-SEM image (A) and EDX maps of **Fe-SS** support

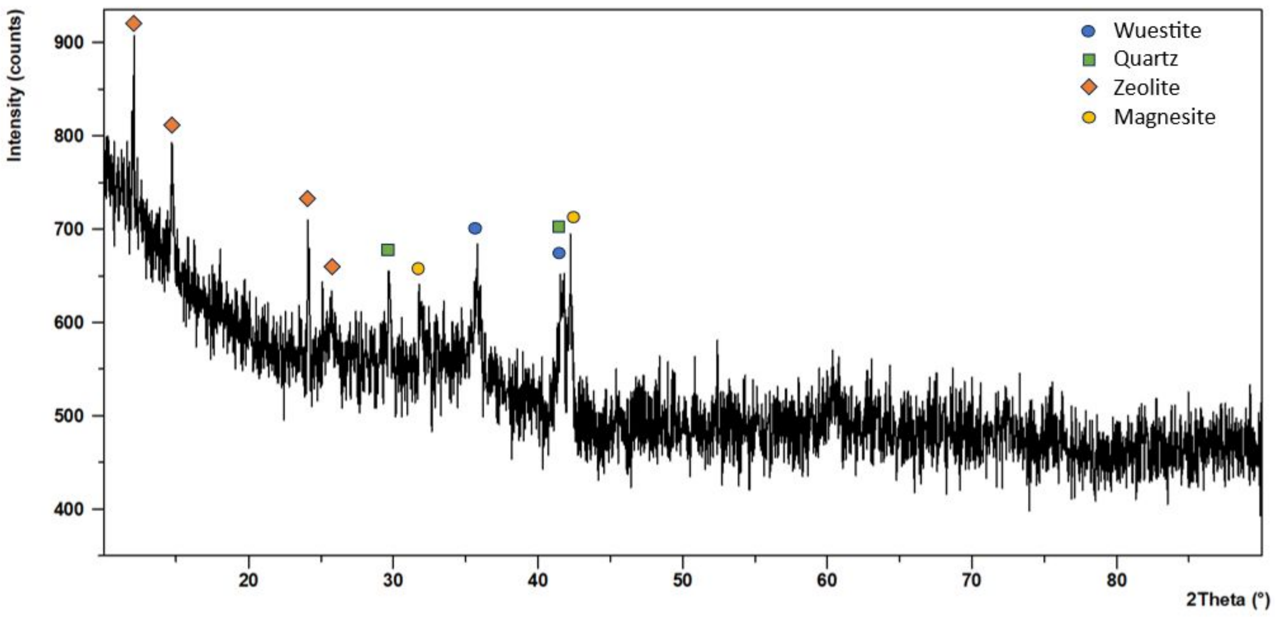

Figure S3 - XRD diffractogram of the **Fe-SS** support

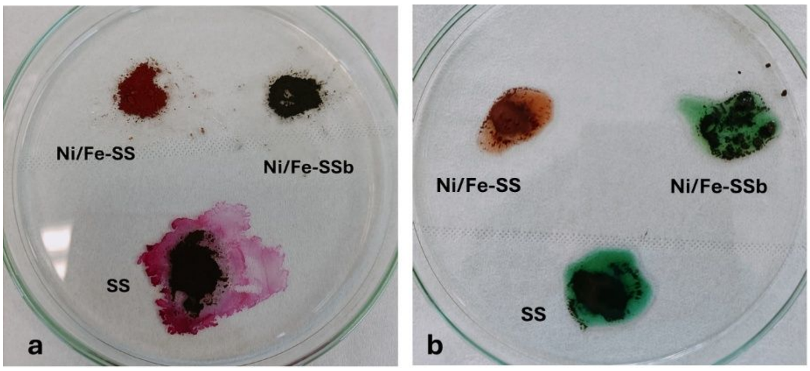

Figure S4 - Hammett test results for **Ni/Fe-SS**, **Ni/Fe-SSb**, and SS (a) using phenolphthalein as an indicator, (b) with a methyl red and bromocresol blue mixture.

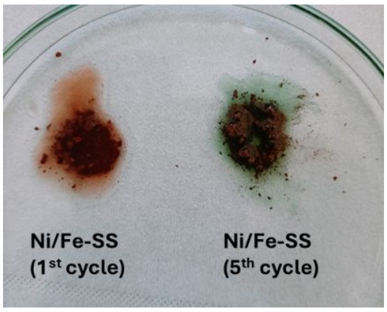

Figure S5 - Hammett test results on the **Ni/Fe-SS** catalyst after the first and fifth reaction cycles using a methyl red and bromocresol blue mixture.

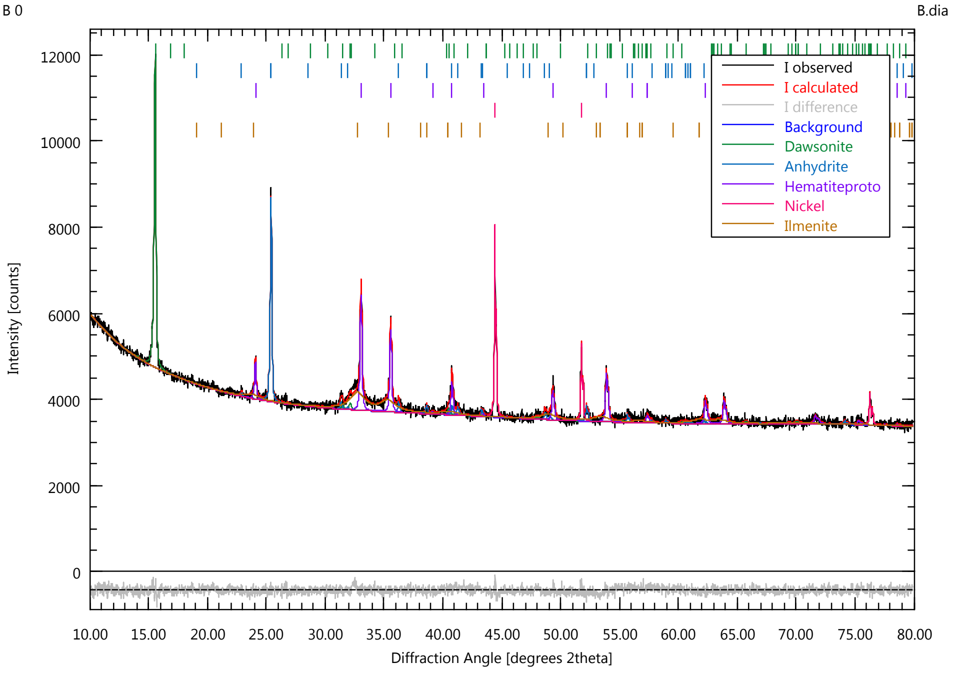

Figure S6 - XRD diffractogram of the **Ni/Fe-SS** before use in catalysis

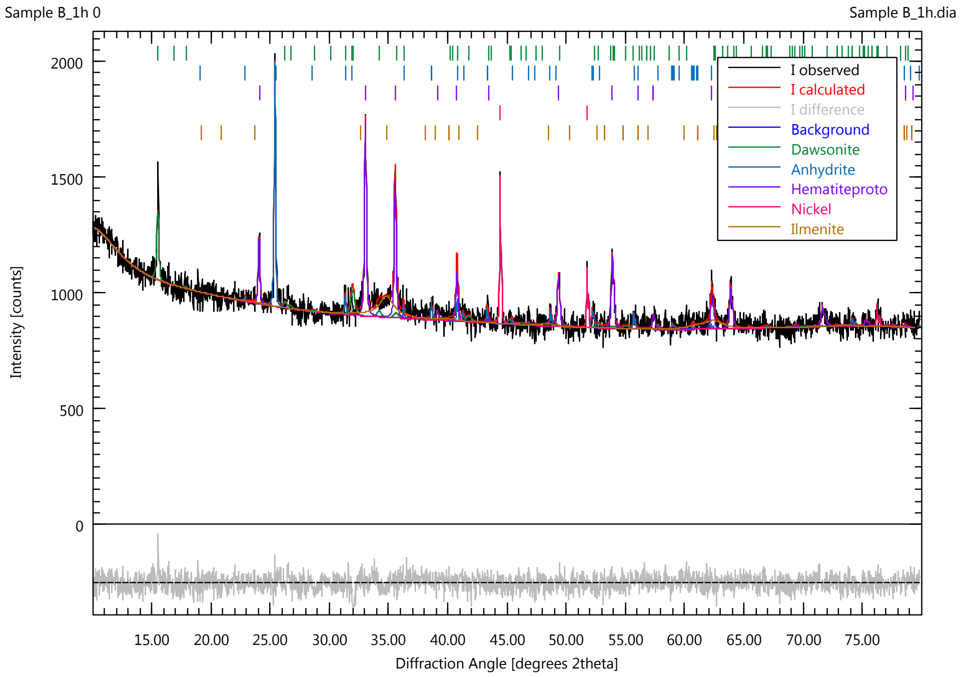

Figure S7 - XRD diffractogram of the **Ni/Fe-SS** after use in catalysis

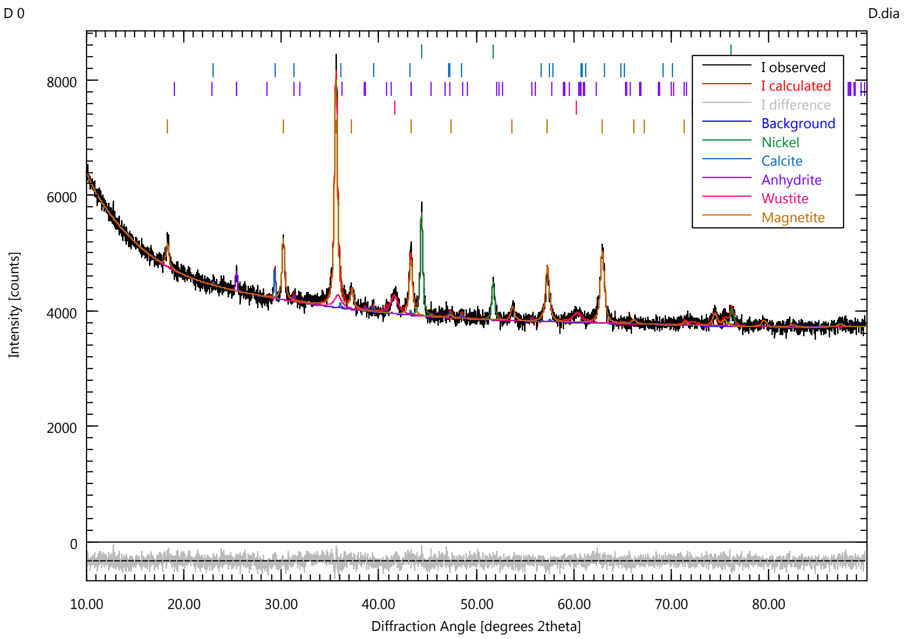

Figure S8 - XRD diffractogram of the **Ni/Fe-SSb** before use in catalysis

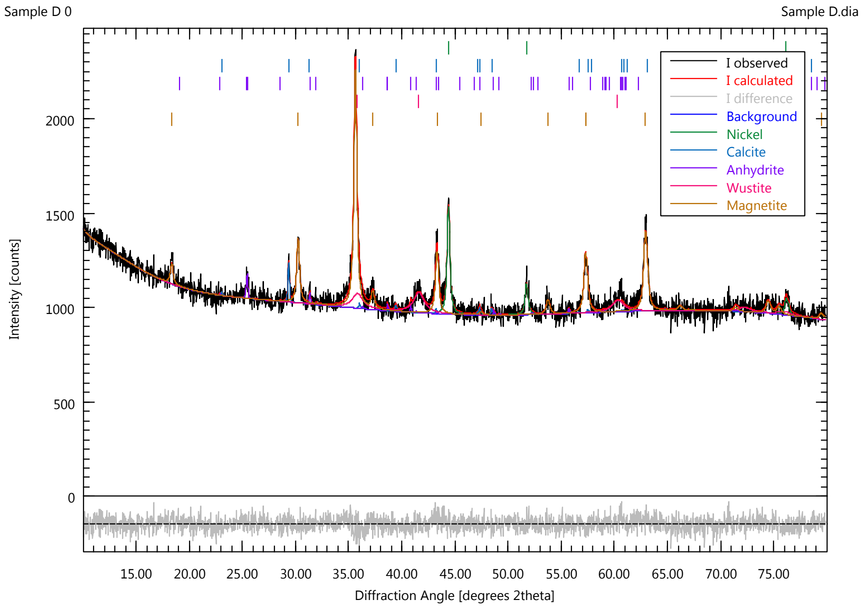

Figure S9 - XRD diffractogram of the **Ni/Fe-SSb** after use in catalysis

Table S1.
Recyclability tests using dihydrogen as the reducing agent and **Ni/Fe-SS** as catalyst.[a]

Entry	Run	C18:0[b] (%)	C18:2[b] (%)	C18:1[b] (%)	CFPP[c] (°C)	OS[c] (h)	IV[c] (g/100g)
1	1st	6.3	15.3	78.4	–5.4	10.7	93.0
2	2nd	7.7	11.1	81.2	–3.3	13.8	87.6
3	3rd	6.8	28.1	65.1	–4.7	7.0	103.6
4	4th	5.8	37.7	56.5	–6.1	5.9	112.3
5	5th	4.8	44.3	50.9	–7.1	5.5	116.4

[a] Reaction conditions: 50 mg of catalyst, 125 mg of biodiesel, 5.0 mL of methanol, 5 bar dihydrogen, 70 °C.
[b] Values reported represent the relative molar percentages of C18 esters (C18:1, C18:2, and C18:0).
[c] Key parameters biodiesel values according to REGULATION (EU) 2022/2383; the threshold established by European standards are: CFPP <10 °C; OS>8 h; IV<120g/100g. Values in red are outside these limits.

Table S2.
Recyclability tests using $NaBH_4$ as the reducing agent and **Ni/Fe-SS** as catalyst[a]

Entry	Run	C18:0[b] (%)	C18:2[b] (%)	C18:1[b] (%)	CFPP[c] (°C)	OS[c] (h)	IV[c] (g/100g)
1	1st	9.7	11.0	79.3	–0.24	14.0	85.7
2	2nd	12.6	15.5	71.9	4.3	10.9	84.4
3	3rd	12.8	22.7	64.5	4.8	8.3	89.7
4	4th	16.4	26.8	56.8	9.8	7.5	90.0
5	5th	15.1	28.5	56.4	8.0	7.2	91.6

[a] Reaction conditions: 100 mg of catalyst, 0.26 mmol $NaBH_4$ 250 mg of biodiesel, 10.0 mL of methanol, RT, t = 20 min.
[b] Values reported represent the relative molar percentages of C18 esters (C18:1, C18:2, and C18:0).
[c] Key parameters biodiesel values according to REGULATION (EU) 2022/2383; the threshold established by European standards are: CFPP <10 °C; OS>8 h; IV<120g/100g. Values in red are outside these limits.

Table S3.
Recyclability tests using dihydrogen as the reducing agent and **Ni/Fe-SSb** as catalyst.[a]

Entry	Run	C18:0[b] (%)	C18:2[b] (%)	C18:1[b] (%)	CFPP[c] (°C)	OS[c] (h)	IV[c] (g/100g)
1	1st	7.7	11.1	81.2	–3.3	13.8	87.6
2	2nd	3.9	43.0	53.1	–8.5	5.6	116.3
3	3rd	4.1	46.8	49.1	–8.5	5.3	121.1

[a] Reaction conditions: 50 mg of catalyst, 125 mg of biodiesel, 5.0 mL of methanol, 5 bar dihydrogen, 70 °C, t = 6 h.
[b] Values reported represent the relative molar percentages of C18 esters (C18:1, C18:2, and C18:0).
[c] Key parameters biodiesel values according to REGULATION (EU) 2022/2383; the threshold established by European standards are: CFPP <10 °C; OS>8 h; IV<120g/100g. Values in red are outside these limits.

Table S4.
Recyclability tests using $NaBH_4$ as the reducing agent and **Ni/Fe-SSb** as catalyst[a]

Entry	Run	C18:0[b] (%)	C18:2[b] (%)	C18:1[b] (%)	CFPP[c] (°C)	OS[c] (h)	IV[c] (g/100g)
1	1nd	13.5	16.9	69.6	5.6	10.2	84.8
2	2rd	10.2	36.6	53.2	1.0	6.1	103.5
3	3th	10.3	37.4	52.3	1.3	6.0	106.5

[a] Reaction conditions: 100 mg of catalyst, 0.26 mmol $NaBH_4$, 250 mg of biodiesel, 10.0 mL of methanol, RT, t = 20 min.
[b] Values reported represent the relative molar percentages of C18 esters (C18:1, C18:2, and C18:0).
[c] Key parameters biodiesel values according to REGULATION (EU) 2022/2383; the threshold established by European standards are: CFPP <10 °C; OS>8 h; IV<120g/100g. Values in red are outside these limits.
